# Targeted bile acid profiles reveal the liver injury amelioration of Da-Chai-Hu decoction against ANIT- and BDL-induced cholestasis

**DOI:** 10.3389/fphar.2022.959074

**Published:** 2022-08-19

**Authors:** YueHua Zhou, YunZhong Zhou, YiFei Li, Wei Sun, ZhaoLong Wang, Long Chen, Ye He, XiaoLong Niu, Jialiang Chen, Guangtao Yao

**Affiliations:** ^1^ Shanghai Innovation Center of TCM Health Service, Shanghai University of Traditional Chinese Medicine, Shanghai, China; ^2^ Institute of Pharmaceutical Preparation Research, Jinghua Pharmaceutical Group Co., Ltd., Jiangsu, China; ^3^ Center for Drug Safety Evaluation and Research, Innovation Research Institute of Traditional Chinese Medicine, Shanghai University of Traditional Chinese Medicine, Shanghai, China; ^4^ Experimental Center for Science and Technology, Shanghai University of Traditional Chinese Medicine, Shanghai, China

**Keywords:** Da-Chai-Hu decoction, intrahepatic cholestasis, extrahepatic cholestasis, targeted metabolomics, bile acid profiles

## Abstract

Multiple types of liver diseases, particularly cholestatic liver diseases (CSLDs) and biliary diseases, can disturb bile acid (BA) secretion; however, BA accumulation is currently seen as an important incentive of various types of liver diseases’ progression. Da-Chai-Hu decoction (DCHD) has long been used for treating cholestatic liver diseases; however, the exact mechanisms remain unclear. Currently, our study indicates that the liver damage and cholestasis status of the α-naphthylisothiocyanate (ANIT)-induced intrahepatic cholestasis and bile duct ligation (BDL)-induced extrahepatic cholestasis, following DCHD treatment, were improved; the changes of BA metabolism post-DCHD treatment were investigated by targeted metabolomics profiling by UPLC-MS/MS. DCHD treatment severely downregulated serum biochemical levels and relieved inflammation and the corresponding pathological changes including necrosis, inflammatory infiltration, ductular proliferation, and periductal fibrosis in liver tissue. The experimental results suggested that DCHD treatment altered the size, composition, and distribution of the BAs pool, led the BAs pool of the serum and liver to sharply shrink, especially TCA and TMCA, and enhanced BA secretion into the gallbladder and the excretion of BAs by the urinary and fecal pathway; the levels of BAs synthesized by the alternative pathway were increased in the liver, and the conjugation of BAs and the pathway of BA synthesis were actually affected. In conclusion, DCHD ameliorated ANIT- and BDL-induced cholestatic liver injury by reversing the disorder of BAs profile.

## Introduction

Cholestasis is a clinical syndrome caused by disturbances of bile secretion, intake, and flow, which cause an accumulation of bile constituents in the liver and serum (van Golen et al., 2018). Cholestasis is not only a common pathological state of many liver diseases but also a key cause of aggravating the liver disease process (Expert Committee of expert consensus on the diagnosis and treatment of cholestatic liver disease, 2015). Various causes may contribute to this condition, including noxious compounds, viral hepatitis, obstruction of the bile duct, genetic abnormality, and disturbance of the intestinal microbiota (Mariotti et al., 2018). A result of this process can be the retention of BAs, accompanied by hepatocyte damage or cholangiocyte injury, inflammation, and apoptosis. In the absence of timely treatment, cholestasis can advance to hepatic failure, fibrosis, cirrhosis, and even hepatic carcinoma (Boyer, 2007). Therefore, reducing hepatic BA overload is a primary goal for treating or blocking the development of the disease. Based on different cholestatic sites, different types can be distinguished, such as the bile duct function in intrahepatic cholestasis patients is basically normal, and the lesions are mainly manifested as lobular bile ducts and are above the bile ducts or hepatocellular lesions (Bacon et al., 2006; Burt et al., 2012). However, extrahepatic cholestasis is usually accompanied by bile duct dysfunction, and the lesions are mainly located in the septal bile ducts and below the bile duct lesions or obstruction (Bacon et al., 2006; Ofliver, 2009). Different types of cholestatic liver disease exist, such as primary biliary cholangitis (PBC), primary sclerosing cholangitis (PSC), intrahepatic cholestasis of pregnancy (ICP), and drug-induced liver injury (DILI). Most patients with cholestatic disease are characterized by intrahepatic cholestasis, but some patients have both intrahepatic and extrahepatic cholestasis (Bacon et al., 2006; European Association for the Study of the Liver, 2009). PBC and PSC are two commonly recognized types of cholestatic liver disease and have a prevalence ranging from 2 to 40 per 100,000 inhabitants and 0 to 16 per 100,000 inhabitants; the former belongs to the category of intrahepatic cholestasis; however, the latter is a mixture of intrahepatic and extrahepatic cholestasis (Boonstra et al., 2012; Nguyen et al., 2014). The development of new drugs to treat cholestasis is greatly hampered by the complications of its etiology and mechanism of injury so that there are few effective therapeutic approaches for cholestasis at present (Chinese Society of Hepatology, Chinese Medical Association; Chinese Society of Gastroenterology, Chinese Medical Association; Chinese Society of Infectious Diseases, Chinese Medical Association, 2016). In clinics, only ursodeoxycholic acid (UDCA) and obeticholic acid (OCA) are approved by the FDA for use in treating cholestasis. UDCA is the most commonly used agent to treat cholestasis, but 40% of patients exhibited tolerance for monotherapy of UDCA (Chascsa et al., 2017). Thus, OCA has gained clinical approval for a new supplementary agent for patients who were unresponsive to UDCA treatment (Samur et al., 2017). However, there are still a range of unmet therapeutic needs.

Currently, there has been an important need to develop novel safe and effective drugs for the treatment of cholestasis; traditional herbal medicines including Chinese materia medica formulae are a potential source of cholestatic treatment drugs. Da-Chai-Hu decoction (DCHD) has been used for treating digestive system diseases for more than 1,000 years in China as classical traditional Chinese materia medica formulae (Qian et al., 2016). Several studies have reported that DCHD exhibits beneficial effects on protecting the liver, cholagogic action, anti-inflammation, and regulating glucose and lipid metabolism (Kuo et al., 2019; Song et al., 2019; Wang et al., 2022). DCHD has been widely used in the clinical treatment of the digestive system diseases including cholecystitis, cholelithiasis, pancreatitis, and gastric and duodenal ulcers (Xue, 2017; Wang and Cui, 2021). It has been shown to exhibit beneficial effects on cholestatic liver damage, significantly reduce the serum levels of ALP, TBA, and TBIL, and alleviate the degree of liver fibrosis and damage (Ohta et al., 1995; Li et al., 2008; Pang et al., 2008). Furthermore, it has been demonstrated that DCHD could decrease the level of FXR mRNA expression in liver tissue and increase TBA levels in the gallbladder (Lu et al., 2015; Wang et al., 2022). A recent study indicated that DCHD inhibited liver inflammation and bile accumulation by activating PPARα, thus preventing acute intrahepatic cholestasis (Xu et al., 2022). However, it remains unclear how DCHD can improve the disorder of BA metabolism and the subsequent pathological changes in the treatment of cholestasis. Recently, studies have highlighted that the functions of BAs not only stimulated the circulatory flow of bile to promote lipid absorption but also acted as signaling molecules to regulate the BA synthesis and the homeostasis of glucose, lipid, and energy metabolism (Arab et al., 2017). A variety of enzymatic reactions and the action of intestinal flora led to a wide variety of BAs; differences in biological properties of BAs including their choleretic effect, solubilization action, and activation of BA receptors are determined by structural differences (Carey, 1984; Cabrera et al., 2019; Fiorucci et al., 2021). Interestingly, BAs and their derivatives are currently the main therapeutic drugs for the treatment of liver metabolic diseases, such as TUDCA and NorUDCA, which are the derivatives of UDCA; OCA is the new semisynthetic BA derivative of CDCA. There is a delicate balance between the therapeutic and damaging effects of BAs; therefore, the size and composition alteration of the BAs pool may reflect the impaired state of BA synthesis due to liver injury, obstruction of bile ducts, or inflammation (Chiang and Ferrell, 2020). Thus, it is conceivable that the work exploring DCHD in the treatment of cholestasis through the direction of BA metabolism has beneficial effects.

The main goal of this study was to examine the hepatoprotective effect of DCHD against acute cholestatic liver damage and to better understand these mechanisms. For this purpose, we established an intrahepatic cholestatic mouse model using ANIT-induced cholestasis and an extrahepatic cholestasis mouse model using BDL-induced liver injury and then researched the conventional serological biochemical and histological changes. The changes of BA metabolism post-DCHD treatment were investigated by targeted metabolomics profiling.

## Materials and methods

### Reagents

The DCHD fluid extract (lot number: 43201101) was provided by Jinghua Pharmaceutical Group Co., Ltd. (Jiangsu, China). The quality control of DCHD in accordance with the Chinese State Food and Drug Administration national standard (YBZ00102008) and the quality control report of DCHD used in this study are shown in the Supplementary Material. According to the national standard, eight traditional Chinese herbs comprise 12 g Bupleuri Radix (Apiaceae; *Bupleurum falcatum* L), 9 g Scutellariae Radix (Lamiaceae; *Scutellaria baicalensis* Georgi), 9 g Paeoniae Radix Alba (Pall Paeoniaceae; *Paeonia lactiflora*), 9 g Pinelliae Rhizoma [Araceae; *Pinellia ternata* (Thunb.) Makino], 9 g Aurantii Fructus Immaturus (Rutaceae; *Citrus × aurantium* L), 6 g Rhei Radix et Rhizome (Polygonaceae; *Rheum palmatum* L), 15 g Zingiberis Rhizoma Recens (Zingiberaceae; *Zingiber officinale* Roscoe), and 12 g Jujubae Fructus (Rhamnaceae; *Ziziphus jujuba* Mill) and were processed in accordance with the standardized production process. DCHD contained paeoniflorin, aloe-emodin, rhein, emodin, chrysophanol, physcion, naringin, hesperidin, neohesperidin, and baicalin, analyzed by HPLC-DAD (Hu Y. et al., 2013; Hu Y. F. et al., 2013; Mao et al., 2017).

ANIT was acquired from Sigma-Aldrich (N4525, Sigma-Aldrich, United States). Ursodeoxycholic acid (UDCA) was acquired from Losan Pharma GmbH (H20150398); the γ-glutamyl transpeptidase ELISA assay kit was obtained from Signalway Antibody (EK3281, SAB, USA). ALT, AST, ALP, TBA, TBIL, and DBIL assay kits were purchased from Shino-Test Corporation (Japan). The total listing of reagents and antibodies used in this study is spelled out in Supplementary Material.

### Animal experiments

All animal protocols were approved by the Use of Live Animals for Teaching and Research Committee of the Shanghai University of Traditional Chinese Medicine (Registration number: PZSHUTCM201120009). Male C57BL/6 J mice (6–8 weeks of age, 20 ± 2 g) were purchased from Vital River Laboratories (Zhejiang, China, Animal License: No. SCXK (Zhejiang) 2019-0001). Mice were raised in the SPF-level breeding room at 22°C with the light/dark cycle (12 h light/12 h dark), provided free access to normal chow diet and sterile water.

**FIGURE 1:**
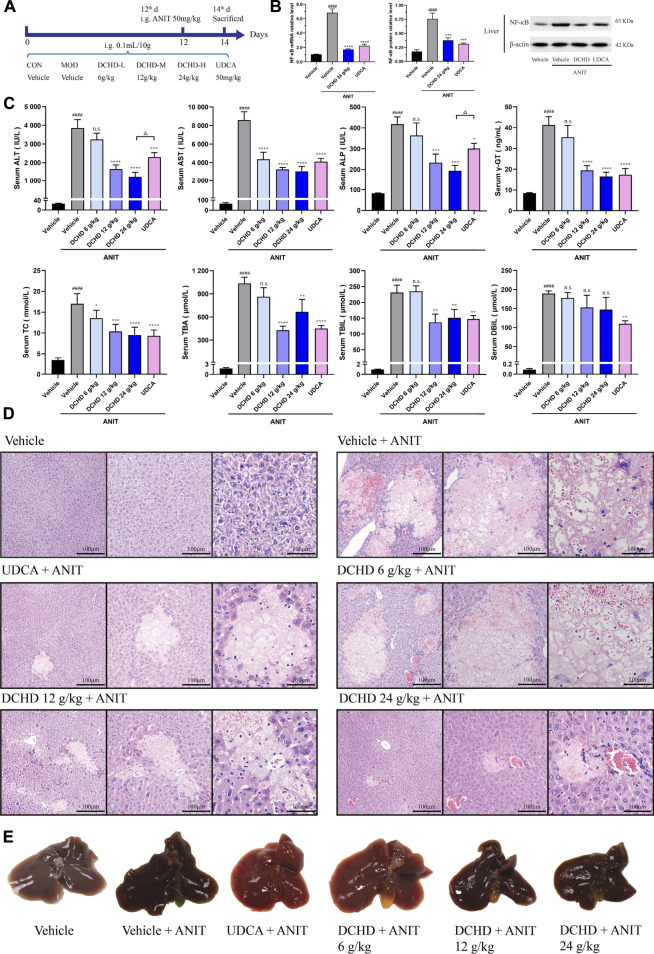
DCHD treatment alleviated cholestatic liver damage and the cholestasis status of the ANIT-induced intrahepatic cholestatic model mice. **(A)** Experimental design demonstrating DCHD treatment of ANIT-induced intrahepatic cholestatic mice. **(B)** Effects of DCHD treatment on hepatic inflammatory cytokine mRNA and protein expression levels in ANIT-induced intrahepatic cholestatic mice. **(C)** Changes of serum biochemical levels in the ANIT-induced intrahepatic cholestatic mice after DCHD treatment. **(D)** Representative images of H&E staining of liver sections in ANIT mice. **(E)** Representative liver and gallbladder pictures. Data are presented as mean ± SEM (*n* = 8). ^#^ *p* < 0.05, ^##^ *p* < 0.01, ^###^ *p* < 0.001, and ^####^ *p* < 0.0001, vs. the vehicle group; ^*^ *p* < 0.05, ^**^ *p* < 0.01, ^***^ *p* < 0.001, and ^****^ *p* < 0.0001, vs. the ANIT group; ^△^ *p* < 0.05, ^△△^ *p* < 0.01, ^△△△^ *p* < 0.001, and ^△△△△^ *p* < 0.0001, vs. the UDCA group.

To determine the protective function of DCHD against the ANIT-induced intrahepatic cholestasis, mice were randomly divided into six groups. (A) The ANIT model group (vehicle + ANIT, *n* = 8), where the mice were fed with ANIT olive oil solution; (B) the control group (vehicle, *n* = 8), where the mice were orally administered with the same volume of olive oil; (C) the high/medium/low dose of DCHD-treated group (DCHD 24 g/kg + ANIT, DCHD 12 g/kg + ANIT, and DCHD 6 g/kg + ANIT; *n* = 8), where the mice were given the same volume of DCHD, and the DCHD dose in this experiment refers to the crude drug dose; and (D) the UDCA-treated group (UDCA 50 mg/kg + ANIT, *n* = 8), where the mice were given the same volume of UDCA. The experimental flow is shown in Figure 1A; the animals were pretreated with vehicle, DCHD, and UDCA everyday by gavage administration for 12 days prior to ANIT induction. After ANIT processing, mice were gavaged with DCHD, UDCA, or vehicle control for another 3 days. The fecal matter and urine were collected using metabolic cages before the animals were euthanized.

**FIGURE 2:**
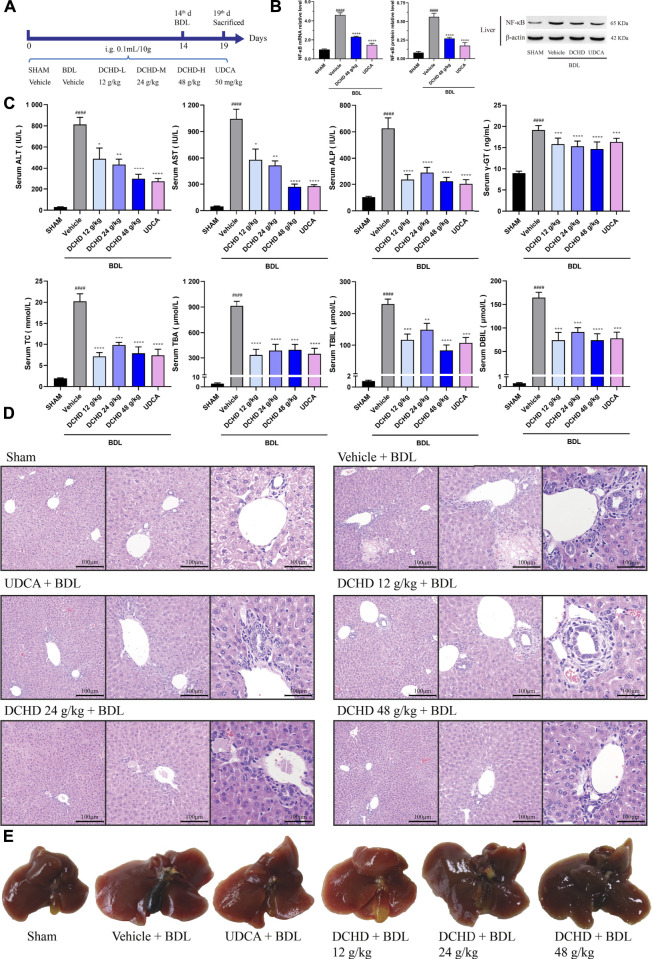
DCHD treatment alleviated cholestatic liver damage and cholestasis status of the BDL-induced extrahepatic cholestatic model mice. **(A)** Experimental design demonstrating DCHD treatment of BDL-induced extrahepatic cholestatic mice. **(B)** Effects of DCHD treatment on hepatic inflammatory cytokines mRNA and protein expression levels in BDL-induced intrahepatic cholestatic mice. **(C)** Changes of serum biochemical levels in the BDL-induced extrahepatic cholestatic mice after DCHD treatment. **(D)** Representative images of H&E staining of liver sections in BDL mice. **(E)** Representative liver and gallbladder pictures. Data are presented as mean ± SEM (*n* = 8). ^#^ *p* < 0.05, ^##^ *p* < 0.01, ^###^ *p* < 0.001, and ^####^ *p* < 0.0001, vs. the sham group; ^*^ *p* < 0.05, ^**^ *p* < 0.01, ^***^ *p* < 0.001, and ^****^ *p* < 0.0001, vs. the BDL group; ^△^ *p* < 0.05, ^△△^ *p* < 0.01, ^△△△^ *p* < 0.001, and ^△△△△^ *p* < 0.0001, vs. the UDCA group.

To determine the protective function of DCHD against the BDL-induced extrahepatic cholestasis, mice were randomly divided into six groups. (A) The BDL model group (vehicle + BDL, *n* = 8), where the mice were orally administered with vehicle control; (B) the Sham group (SHAM, *n* = 8), where the mice were given the same volume of vehicle; (C) the high/medium/low dose of DCHD-treated group (DCHD 48 g/kg + BDL, DCHD 24 g/kg + BDL, and DCHD 12 g/kg + BDL; *n* = 8), where the mice were given the same volume of DCHD; and (D) the UDCA-treated group (UDCA 50 mg/kg + BDL, *n* = 8), where the mice were given the same volume of UDCA. The experimental flow is shown in Figure 2A; the animals were pretreated with vehicle, DCHD, and UDCA everyday by gavage for 14 days prior to BDL surgery. Following the BDL surgery, mice were gavaged with DCHD, UDCA, or vehicle control for another 6 days. The fecal matter and urine were collected using metabolic cages before the animals were euthanized.

In both the experiments, the blood collected from the abdominal aorta, the tissues, the fecal matter, and urine were collected and stored at −80°C or fixed in 4% formalin solution and processed for hematoxylin and eosin (H&E) staining for biochemical assay, pathological analysis, and BAs profile analysis.

### Biochemical analyses

Serum biochemical levels including ALT, AST, ALP, TC, TBA, TBIL, and DBIL were quantified using an automated clinical chemistry analyzer (7080, Hitachi, Japan). γ-glutamyl transpeptidase (γ-GT) levels were measured by using enzyme-linked immunoassay kits.

### Pathological analysis

The pathological analysis of the fixed livers tissues was performed by H&E staining, which included hepatocyte degeneration, inflammation, necrosis, ductular proliferation, and fibrosis. According to the semi-quantitative scoring system (Cullen et al., 2016; Schafer et al., 2018; van Golen et al., 2018), hepatic and biliary injuries were scored as follows: grade 0, within normal limits; grade 1, minimal; grade 2, mild; grade 3, moderate; and grade 4, marked. The extent of necrosis and inflammation was scored (hepatocyte necrosis and inflammatory cell infiltration area 0: absent, 1: <25%, 2: 25–50%, 3: 50–75%, and 4: 75–100%). The extent of biliary hyperplasia and fibrosis was scored (increased bile duct profiles around the portal and fibroblast circumferential proliferation around bile duct area 0: absent, 1: <25%, 2: 25–50%, 3: 50–75%, and 4: 75–100%).

### Bile acid profile analysis

The liver, gallbladder, fecal, and urine samples were collected from the mice for total BA measurement. The serum, liver, and gallbladder samples were collected from the mice for total BA profiling, as described. Briefly, standard and internal standard (IS) solutions were prepared in methanol (IS composition: 1 ml each of 480 ng mycophenolic acid; standard solution composition: 0.1 μM each of CA, CDCA, DCA, LCA, UDCA, HDCA, GCA, TCA, GDCA, TDCA, GCDCA, TCDCA, GUDCA, TUDCA, THDCA, TLCA, GLCA, and GHDCA).

The serum and bile samples were diluted with the dilution vehicle. The mouse liver tissue (1 g in 9 ml of PBS) was used for preparation of the liver tissue homogenate; after vortexing for 1 min, the samples were centrifuged at 5,000 rpm for 15 min at 4°C. Next, 50 μL of serum, liver, and bile sample dilute solutions was added to 150 μL of a mixed solution consisting of IS and methanol solutions (v/v = 2:1), which was vortexed and followed by centrifugation at 12,000 rpm for 10 min at 4°C. Finally, 5 μL of the supernatant was detected by UPLC-MS/MS (ACQUITY UPLC: Waters, MA, USA, Quadrupole 5,500: Applied Biosystems, CA, USA) with an ACQUITY BEH C18 column (1.7 μm, 100 mm × 2.1 mm) (Waters, Milford, MA) and analyzed by Analyst software 1.6.3 platform, integration, and BA quantification, according to previous reports (Yang et al., 2008; Li Y. F. et al., 2017).

### Quantitative real-time PCR

According to the manufacturer’s instructions, TRIzol reagent was used to isolate total RNA from liver samples. Total RNA was used for reverse transcription to cDNA using the PrimeScript RT reagent Kit. GAPDH were used as internal controls, and primers for the experiment are listed in Supplementary Tables S2, S3 Real-time PCR was performed by using the SYBR green reaction mixture in the ABI-StepOnePlus Sequence Detection System (Applied Biosystems).

### Western blotting

Total protein was isolated using the RIPA lysis buffer containing the protease inhibitor cocktail from liver tissues. The determination of the total protein concentration was accomplished by using the BCA protein assay kit. Equal amounts of protein samples were separated by 12% SDS-PAGE in a Bio-Rad Mini-PROTEAN system, and the separated proteins were transferred to polyvinylidene fluoride membranes. The membranes were incubated overnight with specific primary antibodies (FXR, BSEP, and NF-κB-p65). After washing and incubation with the secondary antibody, the protein bands were detected by using ECL Western blotting reagents and quantified by optical densitometry and corrected by the values obtained from the β-actin.

### Multivariate data analysis

Analyst Software 1.6.3 was used to analyze and process the mass spectrometric data, calculate the standard curve of each component of BAs, and then calculate the content of each bile acid component of each test sample according to the standard curve. Relative quantification of α-MCA, β-MCA, and TMCA was performed, according to the concentrations of CA and TCA. Then, the bile acid component concentration data were imported into the MetaboAnalyst 5.0 (https://www.metaboanalyst.ca/MetaboAnalyst/ModuleView.xhtml) database for normalization using the internal standard and analyzed by partial least squares discriminant analysis (PLS-DA) and hierarchical clustering heatmap analysis.

### Statistical analysis

All experimental values were calculated for mean ± SEM. All statistical analyses were performed by SPSS 25.0 and GraphPad Prism 8.0. The normality and homogeneity of variance were tested by using the one-sample Kolmogorov–Smirnov test. The statistical significance of the differences was determined by one-way ANOVA, followed by the least significant difference (LSD) test when equal variance was assumed or Dunnett’s *post hoc* test when equal variance was not assumed, and *p* < 0.05 was considered as significant.

## Results

### DCHD treatment ameliorated ANIT-induced intrahepatic and BDL-induced extrahepatic cholestatic liver injuries

In this study, the data indicated that cholestasis and obvious liver damage were observed after ANIT and BDL induction. The serum biochemical levels including ALT, AST, ALP, TC, TBA, TBIL, and DBIL levels were significantly elevated (Figures 1C, 2C). Clinically, ALT and AST levels reflect the damage status of hepatocytes, and the elevated levels of ALP and γ-GT are used as the criteria for judging cholestatic liver disease (Chinese Society of Hepatology, Chinese Medical Association; Chinese Society of Gastroenterology, Chinese Medical Association; Chinese Society of Infectious Diseases, Chinese Medical Association, 2016; Wei, 2016). The serum ALP level can be used as an important indicator for judging disease severity and disease prognosis in specific patients with liver diseases such as PBC and PSC (Shen and Lu, 2016). The level of the γ-GT activity can reflect the degree of hepatobiliary pathology when intrahepatic and extrahepatic bile ducts were blocked (Luo et al., 2014). In addition, the body weight was significantly decreased, and the organ coefficient was significantly increased (Supplementary Figure S2). The H&E staining of liver confirmed the serum biochemical data, and liver histological changes induced by ANIT treatment and BDL surgery were obvious (Figures 1D,E, 2D,E, Supplementary Figure S3). Hepatocyte degeneration, necrosis, inflammatory infiltration, ductular proliferation, and onion skinning periductal fibrosis around the portal area and central vein were observed (Bedossa et al., 2012; Yang et al., 2018; Gijbels et al., 2021). Our observations showed that the ANIT treatment model focused on hepatocyte necrosis, while the BDL surgery model focused on ductular proliferation and periductal fibrosis (Figures 1D, 2D; Supplementary Figure S3). Since inflammation is also another important manifestation of cholestatic liver injury (Li M. et al., 2017), the expression level of NF-κB mRNA and protein was detected (Figure 1B; Figure 2B). Our results demonstrated that the increased expression level of NF-κB induced by ANIT treatment and BDL surgery was obvious (Figures 1B, 2B).

DCHD treated at doses of 12 g/kg and 24 g/kg could ameliorate ANIT-induced cholestatic liver injury; DCHD treated at doses of 12 g/kg, 24 g/kg, and 48 g/kg could ameliorate BDL-induced cholestatic liver injury. Therefore, our results show that the liver damage and cholestasis status of the intrahepatic and extrahepatic cholestasis models, following DCHD treatment, were improved. Moreover, the 12 g/kg and 24 g/kg DCHD treatment could decrease ALT, AST, ALP, γ-GT, TC, TBA, and TBIL levels in the ANIT-induced intrahepatic cholestasis model; the 24 g/kg DCHD-treated group produced a greater lessened effect on ALT and ALP levels than the UDCA-treated group (Figure 1C). When the 12 g/kg, 24 g/kg, and 48 g/kg DCHD treatment could decrease ALT, AST, ALP, γ-GT, TC, TBA, TBIL, and DBIL levels in the BDL-induced extrahepatic cholestasis model, the depressed effect of DCHD on ALT and AST levels positively correlated with the administration dose (Figure 2C). Additional proof of the liver damage state amelioration, following DCHD therapy, was supplied by H&E staining and the detected results of NF-κB (Figures 1B, D, 2B, D).

### DCHD ameliorated the disordered BA homeostasis in intrahepatic and extrahepatic cholestatic mice

**FIGURE 3:**
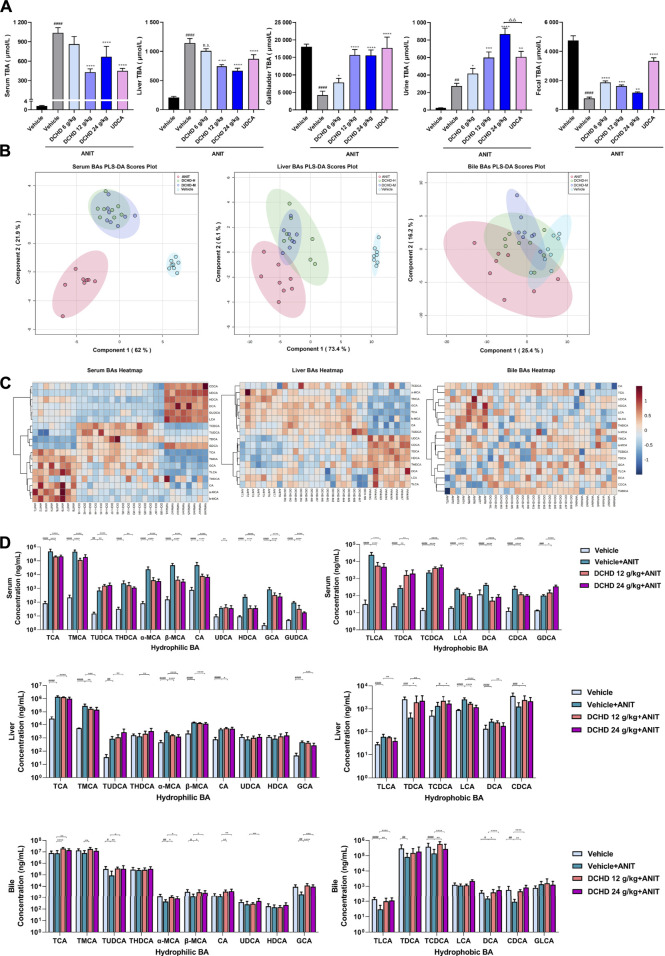
Changes of total bile acid (TBA) and bile acid (BA) profiles in the ANIT-induced intrahepatic cholestatic model mice after DCHD treatment. **(A)** Effects of DCHD treatment on TBA in ANIT-induced intrahepatic cholestatic mice. **(B–C)** Multivariate analysis model (PLS-DA) and hierarchical clustering heatmaps based on the bile acid profile of the serum, liver, and bile. **(D)** Changes of the individual bile acid level in the serum, liver and gallbladder in the ANIT-induced intrahepatic cholestatic model mice after DCHD treatment. Data are presented as mean ± SEM (*n* = 8). ^#^ *p* < 0.05, ^##^ *p* < 0.01, ^###^ *p* < 0.001, and ^####^ *p* < 0.0001, vs. the vehicle group; ^*^ *p* < 0.05, ^**^ *p* < 0.01, ^***^ *p* < 0.001, and ^****^ *p* < 0.0001, vs. the ANIT group; ^△^ *p* < 0.05, ^△△^ *p* < 0.01, ^△△△^ *p* < 0.001, and ^△△△△^ *p* < 0.0001, vs. the UDCA group.

**FIGURE 4:**
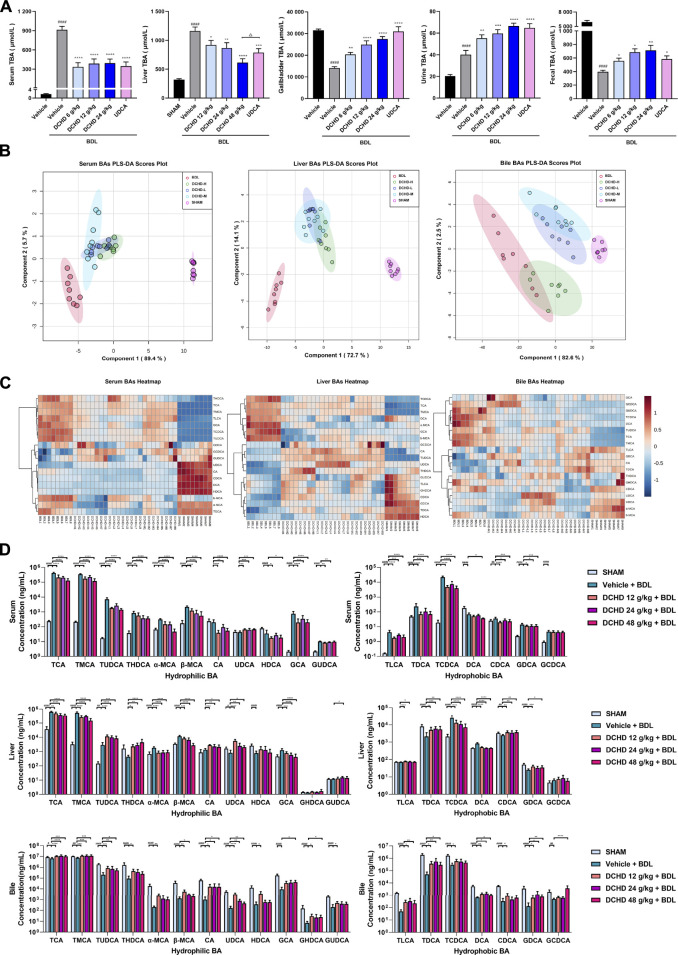
Changes of total bile acid (TBA) and bile acid (BAs) profiles in the BDL-induced extrahepatic cholestatic model mice after DCHD treatment. **(A)** Effects of DCHD treatment on TBA in BDL-induced extrahepatic cholestatic mice. **(B–C)** Multivariate analysis model (PLS-DA) and hierarchical clustering heatmaps based on the bile acid profile of the serum, liver, and bile. **(D)** Changes of the individual bile acid level in the serum, liver, and gallbladder in the BDL-induced extrahepatic cholestatic model mice after DCHD treatment. Data are presented as mean ± SEM (*n* = 8). ^#^ *p* < 0.05, ^##^ *p* < 0.01, ^###^ *p* < 0.001, and ^####^ *p* < 0.0001, vs. the sham group; ^*^ *p* < 0.05, ^**^ *p* < 0.01, ^***^ *p* < 0.001, and ^****^ *p* < 0.0001, vs. the BDL group; ^△^ *p* < 0.05, ^△△^ *p* < 0.01, ^△△△^ *p* < 0.001, and ^△△△△^ *p* < 0.0001, vs. the UDCA group.

Since the BA metabolism disorder has a crucial impact on liver damage induced by cholestatic liver disease, we explored the role of DCHD therapy on BA metabolism in the ANIT and BDL models. The total BA levels of the serum, liver, and urine were significantly increased, and the total BA levels of gallbladder and fecal matter were significantly reduced in the ANIT and BDL models (Figures 3A, 4A). After treatment with DCHD, this situation was reversed except that the increase in the TBA level of urine continued to be enhanced (Figures 3A, 4A). DCHD treatment removed the accumulation of BAs by enhancing BA excretion of the urinary and fecal pathway.

To explore the effects of DCHD on BA metabolism in intrahepatic and extrahepatic cholestasis models *in vivo*, we measured the contents of the 21 types of BAs in the serum, liver, and gallbladder from mice that were given either DCHD or other treatment in this study. The content data on BAs were determined based on PLS-DA and hierarchical clustering heatmap analysis in both intrahepatic and extrahepatic cholestasis models. The BAs profile of DCHD groups was well separated with the vehicle and ANIT group from serum and liver tissues (Figure 3B), and the hierarchical clustering heatmaps of BA composition were different in serum, liver, and gallbladder tissues (Figure 3C). The BAs profile and the hierarchical clustering heatmaps of BA composition of DCHD groups were well separated with the sham and BDL groups from serum and liver tissues (Figures 4B,C), indicating the BA metabolite differences among these groups. The results show that the BA metabolites significantly changed upon ANIT or BDL injury and in response to DCHD treatment. The findings reveal that BA metabolites altered considerably after ANIT or BDL damage, as well as in response to DCHD therapy.

**FIGURE 5:**
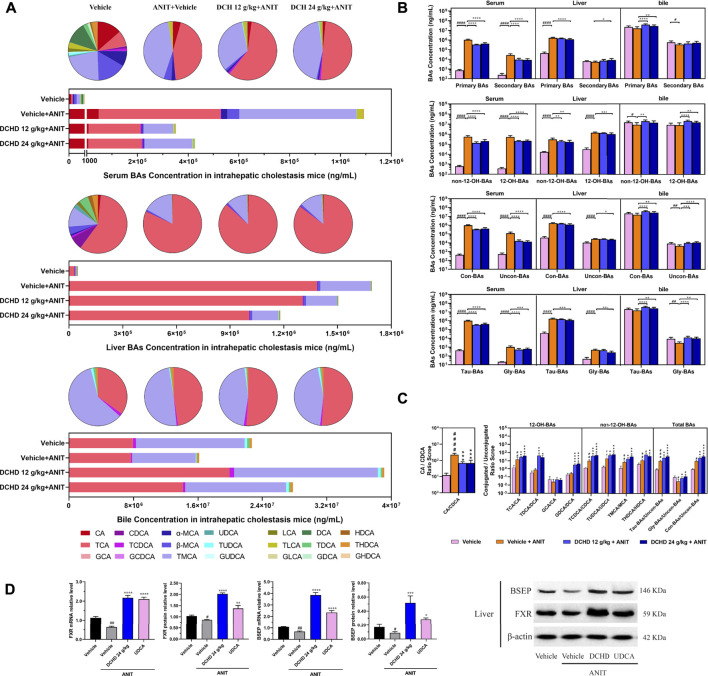
Effects of DCHD treatment on bile acid metabolism in ANIT-induced intrahepatic cholestatic mice. **(A)** Changes of BA composition in ANIT-induced intrahepatic cholestatic mice’s serum, liver, and gallbladder BAs pool upon DCHD treatment. The circle graphs show the mean percentage of the individual bile acids in the BAs pool. The bar graphs show the amount and distribution of the individual BAs. **(B)** Changes of different types of bile acid levels including primary BAs and secondary BAs, 12-OH-BAs (derived from the classical pathway) and non-12-OH-BAs (derived from the alternative pathway), Con-BAs (conjugated BAs) and Uncon-BAs (unconjugated BAs), and Tau-BAs (taurine-conjugated BAs) and Gly-BAs (glycine-conjugated BAs). **(C)** Bar graphs are the ratios of the CA/CDCA and the conjugated/unconjugated BAs in the mouse serum. **(D)** Effects of DCHD treatment on hepatic nuclear receptors, transporter mRNAs, and protein expression levels in ANIT-induced intrahepatic cholestatic mice. Data are presented as mean ± SEM (*n* = 8). ^#^ *p* < 0.05, ^##^ *p* < 0.01, ^###^ *p* < 0.001, and ^####^ *p* < 0.0001, vs. the vehicle group; ^*^ *p* < 0.05, ^**^ *p* < 0.01, ^***^ *p* < 0.001, and ^****^ *p* < 0.0001, vs. the ANIT group; ^△^ *p* < 0.05, ^△△^ *p* < 0.01, ^△△△^ *p* < 0.001, and ^△△△△^ *p* < 0.0001, vs. the UDCA group.

**FIGURE 6:**
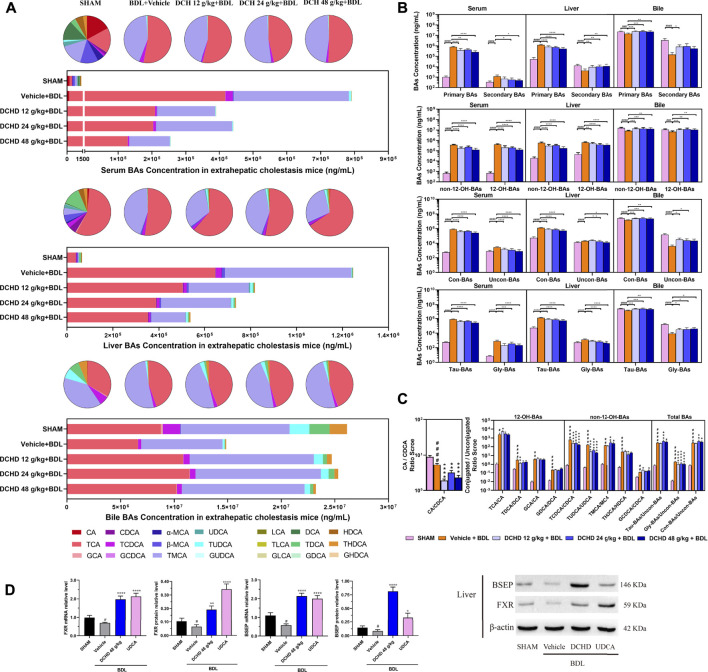
Effects of DCHD treatment on bile acid metabolism in BDL-induced extrahepatic cholestatic mice. **(A)** Changes of BA composition in BDL-induced extrahepatic cholestatic mice’s serum, liver, and gallbladder BAs pool upon DCHD treatment. The circle graphs show the mean percentage of the individual bile acids in the BAs pool. The bar graphs show the amount and distribution of the individual BAs. **(B)** Changes of different types of bile acid levels including primary BAs and secondary BAs, 12-OH-BAs (derived from the classical pathway) and non-12-OH-BAs (derived from the alternative pathway), Con-BAs (Conjugated BAs) and Uncon-BAs (Unconjugated BAs), and Tau-BAs (taurine-conjugated BAs) and Gly-BAs (glycine-conjugated BAs). **(C)** Bar graphs are the ratios of the CA/CDCA and the conjugated/unconjugated BAs in the mouse serum. **(D)** Effects of DCHD treatment on hepatic nuclear receptors, transporter mRNAs, and protein expression levels in BDL-induced extrahepatic cholestatic mice. Data are presented as mean ± SEM (*n* = 8). ^#^ *p* < 0.05, ^##^ *p* < 0.01, ^###^ *p* < 0.001, and ^####^ *p* < 0.0001, vs. the sham group; ^*^ *p* < 0.05, ^**^ *p* < 0.01, ^***^ *p* < 0.001, and ^****^ *p* < 0.0001, vs. the BDL group; ^△^ *p* < 0.05, ^△△^ *p* < 0.01, ^△△△^ *p* < 0.001, and ^△△△△^ *p* < 0.0001, vs. the UDCA group.

To elucidate the distinction further, we investigated the BAs pool size, distribution, and composition including hydrophilic and hydrophobic, primary and secondary, 12-OH (CA and its derivatives) and non-12-OH (CDCA and its derivatives), conjugated and unconjugated, and taurine-conjugated and glycine-conjugated BAs. After ANIT treatment and BDL surgery, the BAs pool size and composition of the serum, liver, and gallbladder from mice significantly changed (Figures 3D, 4D, 5A, 6A). The BAs pool size of the serum and liver was sharply increased, and the BAs pool size of the gallbladder was sharply decreased after ANIT treatment and BDL surgery, while the BAs pool mainly composed of TCA and TMCA was formed in the serum, liver, and gallbladder (Figures 3A, 4A, 5A, 6A). The primary and secondary, 12-OH and non-12-OH, conjugated and unconjugated, and taurine-conjugated and glycine-conjugated BA levels of the serum increased significantly (Figures 5B, 6B). Except for secondary BAs, the changes of other types of bile acids in the liver were consistent with those of the serum, and the change of gallbladder BAs is opposite to that of serum. The ANIT group following treatment with DCHD (12 g/kg and 24 g/kg) and the BDL group following treatment with DCHD (12 g/kg, 24 g/kg, and 48 g/kg); the BAs pool size of the serum and liver was sharply decreased, and the BAs pool size of the gallbladder was sharply increased (Figures 3A, 4A, 5A, 6A). The BAs pool mainly composed of TCA and TMCA still formed in the serum, liver, and gallbladder; the TCA and TMCA levels of the serum and liver were decreased significantly, and the gallbladder TCA and TMCA levels were increased significantly. The levels of BAs synthesized by an alternative pathway including CDCA, TUDCA, and THDCA in the liver were increased significantly, and the DCA level was decreased significantly (Figures 3D, 4D, 5A, 6A). The transformations in primary and secondary, 12-OH and non-12-OH, conjugated and unconjugated, and taurine-conjugated and glycine-conjugated BA levels of the serum, liver, and gallbladder were reversed after DCHD treatment (Figures 5B, 6B). The ratios of each conjugated/unconjugated BA and total conjugated/total unconjugated BAs are shown in Figure 2E. An increase in ratios of total conjugated/total unconjugated BAs and taurine-conjugated/unconjugated BAs was observed in the serum of cholestatic mice after administration with ANIT and BDL surgery; the ratios were further elevated after DCHD intervention (Figures 5C, 6C).

Activation of FXR was shown to provide liver protection in BDL-induced or ANIT-induced cholestatic models (de Aguiar Vallim et al., 2013). BSEP is positively regulated by FXR, and the increased expression of FXR could promote the expression of this protein and accelerate the efflux of BAs from the liver to the capillary bile ducts (Alrefai and Gill, 2007). Consequently, we evaluated the BA nuclear receptor FXR and the transporter BSEP at both the mRNA and protein levels. Hepatic FXR and BSEP mRNA expressions and protein levels were lower in the ANIT and BDL groups. On the contrary, hepatic FXR and BSEP were upregulated by DCHD treatment, either in mRNA or protein expression (Figures 5D, 6D). In summary, these results indicate that BA synthesis was inhibited, and BA efflux was facilitated in the liver after DCHD treatment.

### Difference of the BA spectrum between intrahepatic and extrahepatic cholestatic mice treated with Da-Chai-Hu decoction

In addition to differences in liver tissue damage, the BAs profile also differed between intrahepatic and extrahepatic cholestatic mice. Although the BAs pool of the liver was dominated by TCA and TMCA, the proportions of TCA and TMCA in intrahepatic cholestatic mouse liver tissues reached 81.59% and 16.53%, and the proportions of TCA and TMCA in extrahepatic cholestatic mouse liver tissues reached 51.71% and 44.13% (Figures 5A, 6A, Supplementary). In addition, the CA, α-MCA, β-MCA, and TLCA levels of the serum and the β-MCA levels of the liver from the ANIT model increased significantly and accounted for a significant proportion of their respective BAs pool (Figure 5A). However, the LCA levels of the serum and liver were undetectable; the TCDCA and TUDCA levels of the serum and the TCDCA and β-MCA levels of the liver from the BDL model increased significantly and accounted for a significant proportion of their respective BAs pool (Figure 6A). The HDCA and DCA levels of the serum in the intrahepatic cholestatic mice were significantly increased, while the HDCA and DCA levels of the serum in the extrahepatic cholestatic mice were significantly decreased (Figures 3D, 4D). The changes of TUDCA, TDCA, TUDCA/UDCA, and TCDCA/CDCA levels in intrahepatic and extrahepatic cholestatic model mice were opposite after DCHD intervention (Figures 5C, 6C). The CA levels of the serum and liver in the intrahepatic cholestatic mice were significantly increased, while the CA levels of the serum and liver in the extrahepatic cholestatic mice did not show significant changes, and the CDCA level was significantly increased in the serum of both cholestatic model groups’ mice and significantly decreased in the liver of both cholestatic model groups’ mice (Figures 3D, 4D). The BA ratios could reflect changes of enzymatic processes in BA metabolism; the CA: CDCA ratio was selected to test if a possible shift in BA synthesis from the classical to the alternative BA pathway occurs in the liver (MahmoudianDehkordi et al., 2019). In comparison to the vehicle group, the CA: CDCA ratio of the ANIT group mice was significantly increased, while that of the BDL group mice demonstrated much lower levels than that of the sham group, suggesting that the BA synthesis in the ANIT-induced intrahepatic cholestatic model mice was still synthesized by the classical pathway, while that of BDL-induced extrahepatic cholestatic model mice shifted to the alternative pathway (Figures 5C, 6C). The CA: CDCA ratio of intrahepatic and extrahepatic cholestatic model mice after DCHD intervention was significantly decreased, suggesting that DCHD intervention induced the conversion of the BA synthesis pathway from the classical pathway to the alternative pathway in intrahepatic cholestatic model mice, while in extrahepatic cholestasis model, it further shifts toward alternative pathways (Figures 5C, 6C).

## Discussion

Da-Chai-Hu decoction (DCHD), a classical formula from the *Treatise on Febrile Disease*, is used in the treatment of digestive system diseases such as pancreatitis, cholecystitis, cholelithiasis, and gastric and duodenal ulcers. Previous studies have demonstrated DCHD has a good liver protective effect; the mechanism of DCHD on the protective effect against liver injury caused by cholestasis is still obscure. In this investigation, our results revealed that DCHD has a therapeutic effect on liver injury and cholestasis induced by ANIT and BDL. It was notable that the size, composition, and distribution of the BAs pool were altered with DCHD treatment. DCHD treatment led the BAs pool of the serum and liver shrink and enhanced BA secretion into the gallbladder and excretion of the urinary and fecal pathway. TCA and TMCA, which are more prone to cause inflammation than others, were reduced (Zhang et al., 2011). TUDCA was increased in the liver, and the pathway of BA synthesis was actually affected. However, these changes were likely to inhibit BA synthesis and enhance BA efflux by affecting the FXR signaling pathway, thereby affecting BAs profile changes.

To mimic cholestatic liver damage to examine the mechanism of the cholestatic liver disorder and monitor the effectiveness of innovative treatments, a large number of animal cholestatic models have been developed including surgery, chemical, viral infections, and gene-knockout–induced cholestasis in the last 20 years. α-naphthyl-isothiocyanate (ANIT) and bile duct ligation (BDL) are two frequent and valuable modeling methods but with different mechanisms. ANIT has generally been accepted as prototypic hepatotoxicants for provided acute or chronic cholestatic liver injury model (Desmet et al., 1968). *In vivo*, ANIT undergoes hepatocyte metabolism and forms ANIT-GSH complexes that temporarily lose their toxicity after conjugation with GSH, secreted into bile, whereby free ANIT toxicity recovers and exerts toxic effects on the bile duct epithelial cells (Orsler et al., 1999; Joshi et al., 2017; Yang et al., 2017). Persistent exposure to ANIT causes significant damage and necrosis to the bile ducts and hepatocytes, even periportal inflammation and cholestasis; however, the extrahepatic bile ducts are not damaged; thus, the ANIT-induced model resembles more of the intrahepatic cholestatic liver disease in humans (Mariotti et al., 2018; Gijbels et al., 2021). BDL has generally been accepted as the most widely used model mimicking extrahepatic cholestasis induced by bile duct obstruction. BDL surgery induces strong proliferation of cholangiocytes, resulting in cholestasis, ductular proliferation, and onion skinning periductal fibrosis (Mariotti et al., 2018; Gijbels et al., 2021). In this research, the ALT and AST levels of the ANIT-induced intrahepatic cholestasis model were significantly higher than those of the BDL-induced extrahepatic cholestasis model. Biochemical levels and pathological observations suggest that the ANIT treatment model focused on hepatocyte necrosis, and the BDL surgery model focused on ductular proliferation and periductal fibrosis, which was in agreement with the previous experiment. The relatively high mortality rates are an unavoidable problem; thus, the BDL procedure and the administered dose of DCHD were adjusted as described (Weerachayaphorn et al., 2014; Dong et al., 2021).

The BA secretion can be disrupted by multiple types of liver diseases, especially cholestatic liver diseases and biliary diseases; however, hepatic buildup of BAs is currently thought to be a driving force of various types of liver diseases worsening (Boyer, 2007; Fickert and Wagner, 2017). Consequently, reducing the hepatic BA overload is a primary goal for the cholestatic liver disease therapy. Previous evidence has shown that DCHD treatment reduced the level of BAs in the serum at the same time and effectively promoted the excretion of stones in the biliary tract (Ohta et al., 1995; Li et al., 2008; Pang et al., 2008). Our finding that the levels of TBA of the serum and liver reduced, and the level of gallbladder reduction was consistent with studies recently published. The inconsistency in the results for the TBA level in urine and in feces after ANIT and BDL treatment brought our attention to the adaptive reaction to cholestasis that inhibits renal reabsorption of BAs and enhanced renal excretion of BAs in order to minimize hepatic injury (Soroka et al., 2010; de Aguiar Vallim et al., 2013). It was previously reported that BAs can be released into the blood circulation system through hepatocyte basolateral BA transporter and the cholehepatic shunt pathway (Xia et al., 2006) and then eliminate overload bile acids by renal excretion and adapt to cholestasis induced by bile duct obstruction (Soroka et al., 2010; Li and Chiang, 2014; Li et al., 2018). Our work demonstrates that DCHD administration led to enhancement of BA renal and fecal excretion and minimized hepatic injury, which may be related to the enhanced release of BAs into the circulatory system by DCHD *via* the hepatocyte basolateral BA transporter and eliminate overload bile acids through renal excretion while reducing BA reabsorption in the ileum and colon.

The differences in the BA structure account for differences in their biological properties, so we investigated the effect of DCHD therapy on BA metabolism in the ANIT and BDL models. In the present investigation, we found that the BAs pool size of the serum and liver, especially the TCA and TMCA levels of the serum and liver, was sharply decreased, and the TUDCA and THDCA levels of the liver were increased significantly. This is a critical finding as previous studies reported that taurine-conjugated BAs in mice are more sensitive to the cholestatic state than the other types of bile acid components, and the TCA and TMCA levels of cholestatic model animals increased sharply (Yang et al., 2008; Zhang et al., 2011; Wang et al., 2020; Thibaut et al., 2021). Compared with the direct toxic effects of hydrophobic BAs, the inflammatory injury caused by the massive accumulation of BA components such as TCA, TMCA, and MCA is more obvious (Murphy et al., 2005; Zhang et al., 2011). Evidence shows that the DCHD inhibited expressions of hepatic TNF-α and IL-6 and improved the inflammatory state of the liver (Chang and Wang, 2015). In this study, DCHD treatment led to the diminution of hepatocyte degeneration, necrosis, or inflammatory infiltration, and the lower expression levels of NF-κB mRNA and protein in the liver of ANIT- and BDL-induced mice. The improvement of liver inflammation by DCHD may be interrelated to the inhibition of the inflammatory response pathway and the reduction of TCA and TMCA accumulation. In addition, it was previously reported that DCHD treated cholecystitis and pancreatitis *via* inhibition of breaking a continuous cascade of inflammatory mediators (Lu et al., 2015; Fan, 2019; Wang et al., 2022). Apart from inhibiting the inflammatory response in the digestive system such as the liver, gallbladder, and pancreas, the DCHD also has an improving effect on inflammation in the lung tissue and circulatory system (Yang, 2017; Zhang et al., 2020). TUDCA is a taurine-binding derivative of UDCA, and the bound sulfonic acid group enhances the polarity and hydrophilicity of the molecule which more effectively promotes the hydrophilic transformation of bile pools and protects hepatocytes and cholangiocytes (Invernizzi et al., 1999; Chiang, 2009). At the same time, UDCA can reduce the saturation index of cholesterol in bile and intestinal cholesterol absorption and effectively prevent gallstone deposition (Hardison and Grundy, 1984; Setchell et al., 1996; Dorvash et al., 2018). In the experiment, it could be observed that the level of secondary BAs in the liver of cholestatic mice increases after administration of DCHD, and the increase of such bile acid levels was mainly caused by the increase in HDCA and THDCA levels in the liver. HDCA and THDCA belong to HCA species. Relevant studies have verified that HCA species were correlated with clinical blood glucose markers and played a key role in maintaining glucose homeostasis (Jia et al., 2021; Zheng et al., 2021).

The BA synthesis occurs mainly in the liver *via* two distinct pathways, the classical or neutral pathway, which predominantly produces CA and CDCA. It accounts for about 75% of BA production in the main pathway in the normal condition; the alternative or acidic pathway predominantly produces CDCA (Duane and Javitt, 1999; Schwarz et al., 2001). Cholesterol-7α-hydroxylase (CYP7A1) is the rate-limiting enzyme for BA synthesis, while sterol-12α-hydroxylase (CYP8B1) decides the ratio of CA to CDCA by promoting CA synthesis (Vlahcevic et al., 1991; Chiang, 2017). When the body is in certain pathological states, the activities of CYP7A1 and CYP8B1 in the liver are down-regulated, which regulates the balance of BA metabolism by stimulating the alternative pathway to produce CDCA (Axelson and Sjövall, 1990; Jia et al., 2021). Several research studies have previously been published which demonstrated that the non-12-OH BAs of cholesterol-7α-hydroxylase-deficient or transgenic expression mice increased significantly, which were derived from the alternative pathway, although the BAs pools of the former were significantly decreased, while those of the latter were significantly increased (Li et al., 2010; Ferrell et al., 2016). Targeted disruption of the sterol 12α-hydroxylase gene results in CDCA and MCA synthesized by the alternative pathway was significantly increased, and the CA synthesized by the classical pathway was almost eliminated (Kaur et al., 2015). Recent research studies have emphasized the key role of the non-12-OH-BAs synthesized by the alternative pathway in regulation of lipid, cholesterol, and energy homeostasis (Jia et al., 2021). If the BAs synthesized by the alternative pathway are inhibited, the proportion of 12-OH-BAs synthesized by the classical pathway will increase, which will further lead to a decrease in the liver’s ability to respond to hepatic lipid homeostasis and the inflammatory state, and the degree of liver fibrosis was significantly associated with 12-OH-BAs (Jia et al., 2018; Jia et al., 2021; Xie et al., 2021). Along this line, we calculated the ratio of CA to CDCA because the ratio of CA to CDCA was selected to test if a possible shift in BA synthesis from the classical to the alternative BA pathway occurs in the liver (Chen et al., 2019; MahmoudianDehkordi et al., 2019; Weingartner et al., 2021). In the present investigation, we found that the BA synthesis in the ANIT-induced intrahepatic cholestatic model was still dominated by the classical pathway; however, the BA synthesis pathway in the BDL-induced extrahepatic cholestatic model shifted to the alternative pathway, and the proportion of TCA and TMCA in the two types of model mouse’s liver supported this conclusion. The difference in BA synthesis in both the ANIT-induced intrahepatic cholestatic model and BDL-induced extrahepatic cholestatic model may be due to the different mechanisms of model formation; as mentioned earlier, ANIT causes significant damage and necrosis to the hepatocytes and bile ducts, while BDL causes strong proliferation of cholangiocytes and periductal fibrosis in the early stages of surgery (Mariotti et al., 2018; Gijbels et al., 2021). DCHD treatment promoted the conversion of the bile synthesis pathway from the classical pathway to the alternative pathway in the intrahepatic cholestasis model and further strengthens the transition to the alternative pathway in the extrahepatic cholestatic model.

FXR is a very important part of the precise regulation system of BAs; as an important BA receptor, it is distributed in a variety of tissues. Among them, the expression level of FXR is the highest in the liver and intestine. In these two types of tissues, FXR receptors are deeply involved to regulate BA synthesis and transport to ensure optimal bile pool size and maintain BA metabolic homeostasis (Mencarelli and Fiorucci, 2010; Sonne, 2021). FXR activation was found to inhibit BA synthesis and prevent liver damage caused by excessive BA synthesis. At the same time, FXR activation inhibited hepatic NTCP and intestinal ASBP to reduce hepatic uptake of BAs and intestinal reabsorption of BAs, enhanced expression of hepatic BA transporters such as BSEP, MRP3, and OSTα/β, promoted the BA efflux, and decreased hepatic BA accumulation (Trauner et al., 2017; Stofan and Guo, 2020). Activation of FXR was shown to provide liver protection in BDL-induced or ANIT-induced cholestatic models (de Aguiar Vallim et al., 2013). Our work result was consistent with these findings; treatment with DCHD and the expressions of FXR and BSEP in the cholestatic mouse liver were activated in mice, and hepatic BA accumulation decreased. The activation of FXR in the liver tissue was more inclined to regulate CYP8B1, followed by the inhibition of CYP7A1, so it mainly inhibited the synthesis of CA (Kim et al., 2007; Kong et al., 2012). The CA was an important determinant of intestinal cholesterol absorption, and intestinal cholesterol absorption was suppressed, induced by reducing CA synthesis (Murphy et al., 2005). Of note, serum CA and TC levels in mice with intrahepatic and extrahepatic cholestasis significantly decreased after DCHD intervention. Study results show that DCHD has the potential to treat obesity and non-alcoholic fatty liver disease (Han et al., 2020). Upon DCHD treatment, the triglyceride content in hepatocytes was significantly reduced, and the fat deposition in the liver was reduced (Nakayama et al., 2007; Wang et al., 2021). Studies have pointed out that the *Scutellaria baicalensis* extract is the key component of DCHD to reduce serum total cholesterol levels and inhibit lipase and lipid absorption (Matsuo et al., 2018). Thus, whether DCHD reduces the supply of BA synthesis raw materials by inhibiting intestinal cholesterol absorption deserves further investigation.

## Conclusion

**FIGURE 7:**
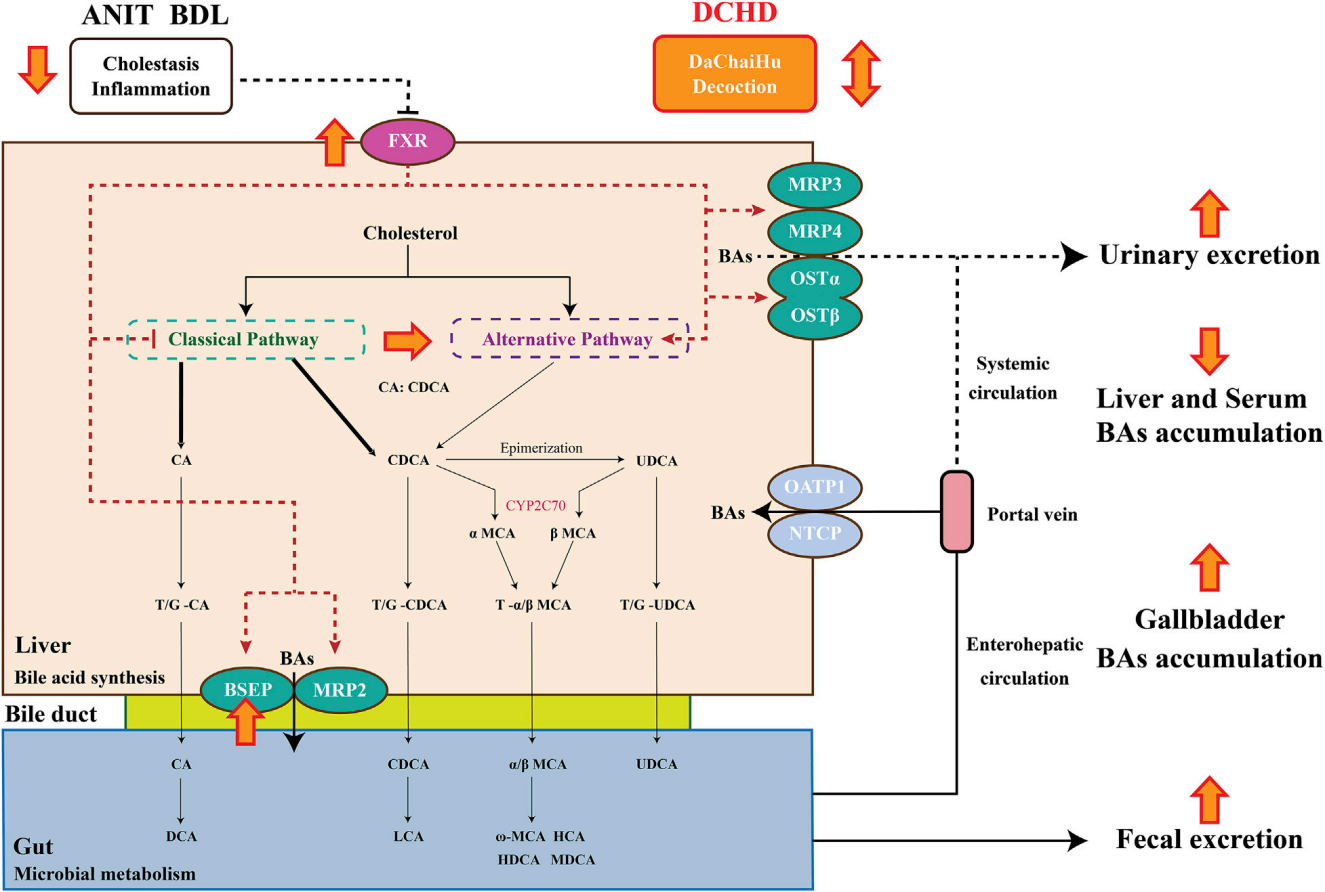
Bile acid synthesis and metabolism and the protective mechanism of Da-Chai-Hu decoction ameliorate acute cholestatic-induced liver injury through reversing the bile acid metabolism disorder.

In conclusion, our current study reports that the liver damage and cholestasis status of the intrahepatic and extrahepatic cholestasis, following DCHD treatment, were improved. The potential mechanism of DCHD on ameliorating cholestatic liver injury was revealed, which involved inhibition of BA synthesis and enhanced BA efflux *via* activation of the FXR signaling pathway, altered the size, composition and distribution of the BAs pool, and regulation of BA synthesis pathways, reducing overload BAs, especially TCA and TMCA in the liver, enhancement of BA fecal and urine elimination pathways, and reversed the imbalance of BA homeostasis (Figure 7). Overall, our findings provide reliable verification of the therapeutic action of DCHD on cholestatic liver injury and provide theoretical and experimental basis for the clinical application and new drug development of DCHD in the treatment of cholestatic liver disease.

## Data Availability

The original contributions presented in the study are included in the article/Supplementary Materials; further inquiries can be directed to the corresponding author.
