# Supplementary material for: Targeted bile acid profiles reveal the liver injury amelioration of Da-Chai-Hu decoction against ANIT- and BDL-induced cholestasis
Source: Front Pharmacol. 2022 Aug 19;13:959074. doi: 10.3389/fphar.2022.959074 (PMC9437253; doi:10.3389/fphar.2022.959074)
Supplement: Supplementary file 1 [file DataSheet1.docx]

***Supplementary Material***

**1. Supplementary Figure**


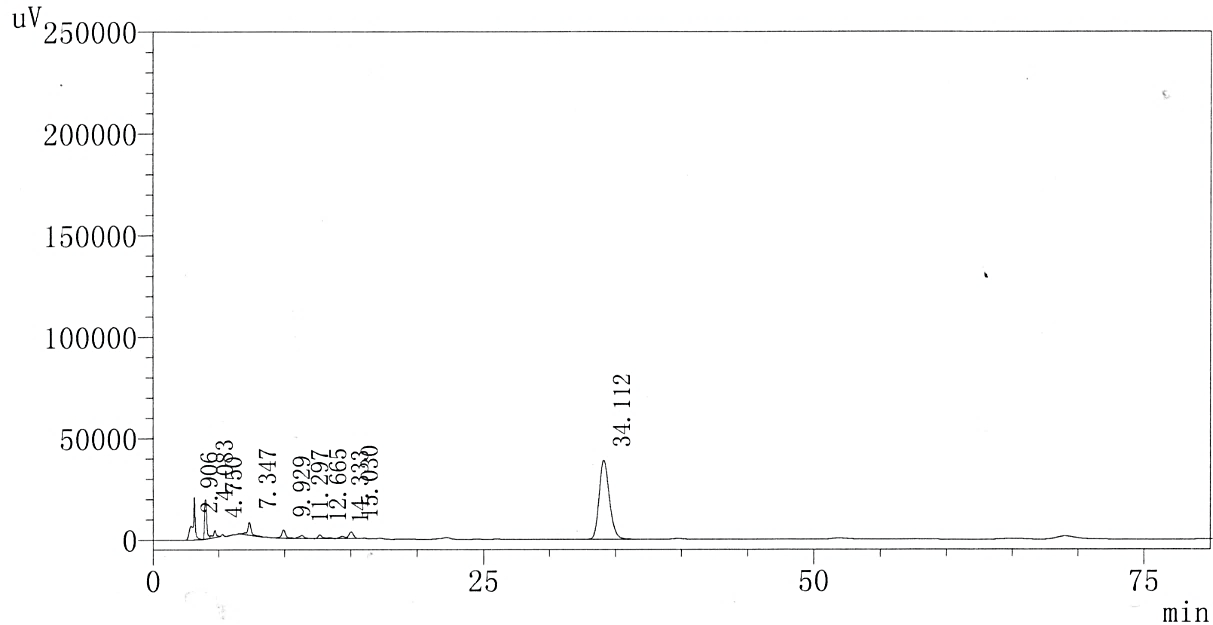


**Supplementary Figure S1**

The HPLC chromatograms of DCHD (lot number:43201101) methanol solution at 317nm, Baicalin was used as a quantitative index.

| Peak | Name | RT [min] | Area | Height | Tailing Factor | Theoretical Plate |
| --- | --- | --- | --- | --- | --- | --- |
| 1 |  | 2.906 | 308633 | 6713 | 1.474 | 324.123 |
| 2 |  | 4.083 | 229324 | 17096 | 0.000 | 3473.956 |
| 3 |  | 4.750 | 55673 | 3153 | 0.000 | 3533.115 |
| 4 |  | 7.347 | 120747 | 5880 | 0.886 | 5089.564 |
| 5 |  | 9.929 | 86754 | 3892 | 1.269 | 6286.084 |
| 6 |  | 11.297 | 40906 | 1485 | 1.059 | 4568.322 |
| 7 |  | 12.665 | 44063 | 1770 | 0.000 | 7496.353 |
| 8 |  | 14.333 | 44795 | 1065 | 0.000 | 4633.747 |
| 9 |  | 15.030 | 81461 | 3298 | 0.000 | 7709.271 |
| 10 | Baicalin | 34.112 | 2103441 | 38911 | 1.205 | 9357.418 |

**
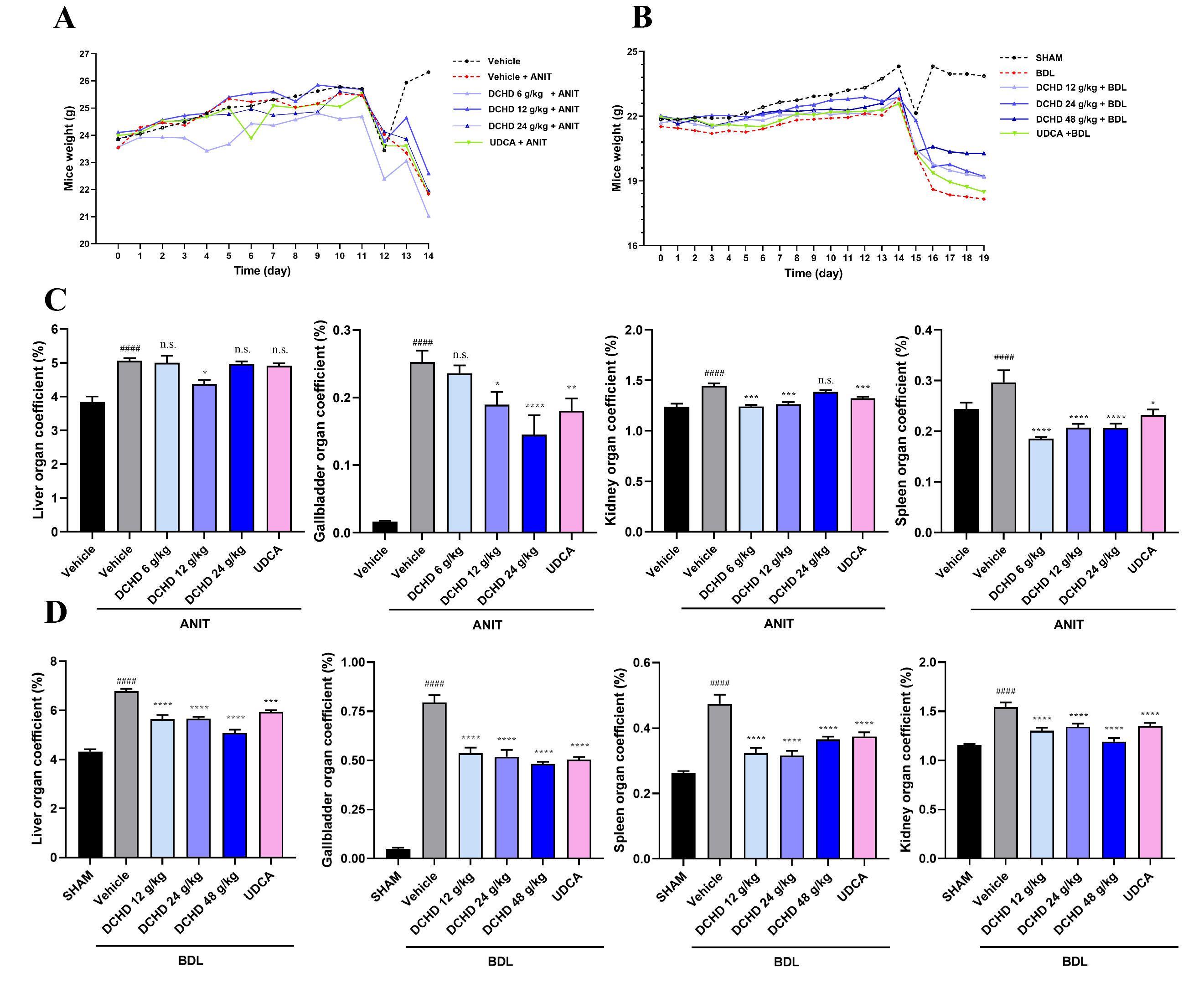
**

**Supplementary Figure S2**

**(A)** The effects of DCHD treatment on body weight in ANIT-induced intrahepatic cholestasis mice. **(B)** The effects of DCHD treatment on body weight in BDL-induced extrahepatic cholestasis mice. **(C)** The effects of DCHD treatment on organ coefficient in ANIT-induced intrahepatic cholestasis mice. **(D)** The effects of DCHD treatment on organ coefficient in BDL-induced extrahepatic cholestasis mice. Data are presented as mean ± SEM (n=8). ^#^P <0.05, ^##^P < 0.01, ^###^P < 0.001, ^####^P < 0.0001, versus the Vehicle or sham group; ^*^P < 0.05, ^**^P < 0.01, ^***^P < 0.001, ^****^P < 0.0001, versus the ANIT or BDL group; ^△^P < 0.05, ^△△^P < 0.01, ^△△△^P < 0.001, ^△△△△^P < 0.0001, versus the UDCA group.


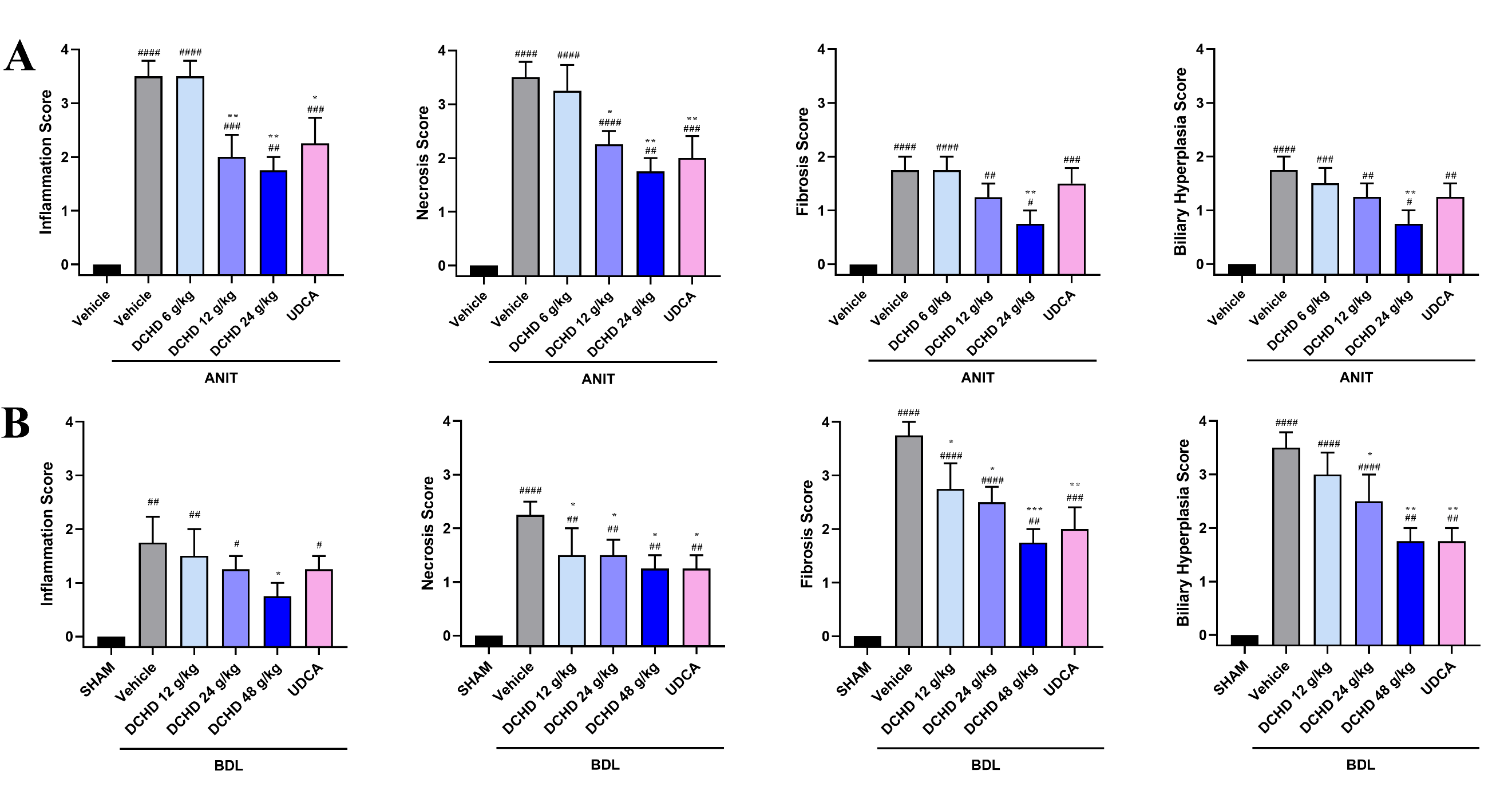


**Supplementary Figure S3**

**(A)** The subjective histopathological scores of liver sections in ANIT-induced intrahepatic cholestasis mice. **(B)** The subjective histopathological scores of liver sections in BDL-induced extrahepatic cholestasis mice. Data are presented as mean ± SEM (n=4). ^#^P <0.05, ^##^P < 0.01, ^###^P < 0.001, ^####^P < 0.0001, versus the versus the Vehicle or sham group; ^*^P < 0.05, ^**^P < 0.01, ^***^P < 0.001, ^****^P < 0.0001, versus the ANIT or BDL group.


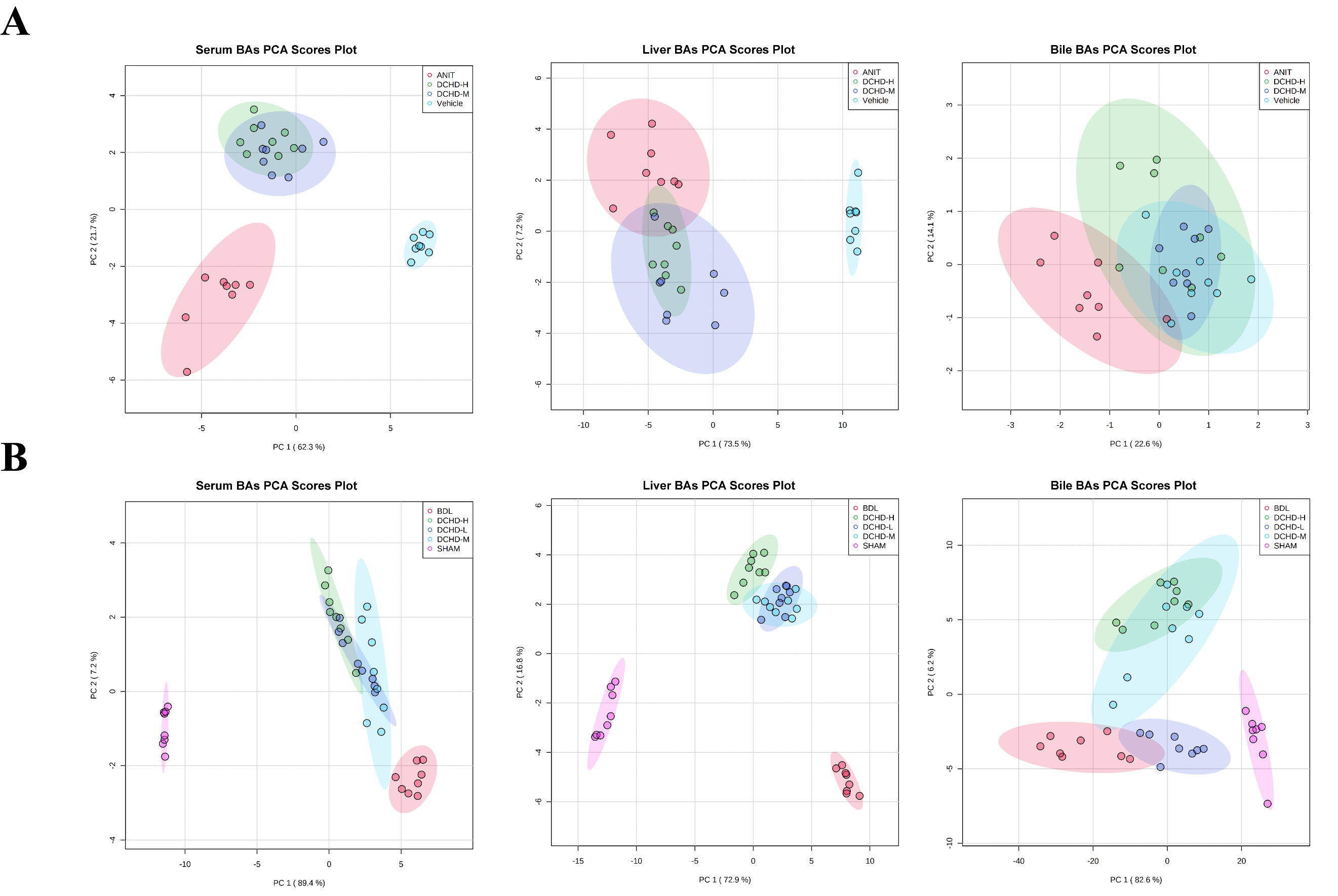


**Supplementary Figure S4**

**(A)** The Principal Component Analysis (PCA) based on the bile acid profile of the serum, liver, bile in ANIT-induced intrahepatic cholestasis mice. **(B)** The Principal Component Analysis (PCA) based on the bile acid profile of the serum, liver, bile in BDL-induced extrahepatic cholestasis mice.


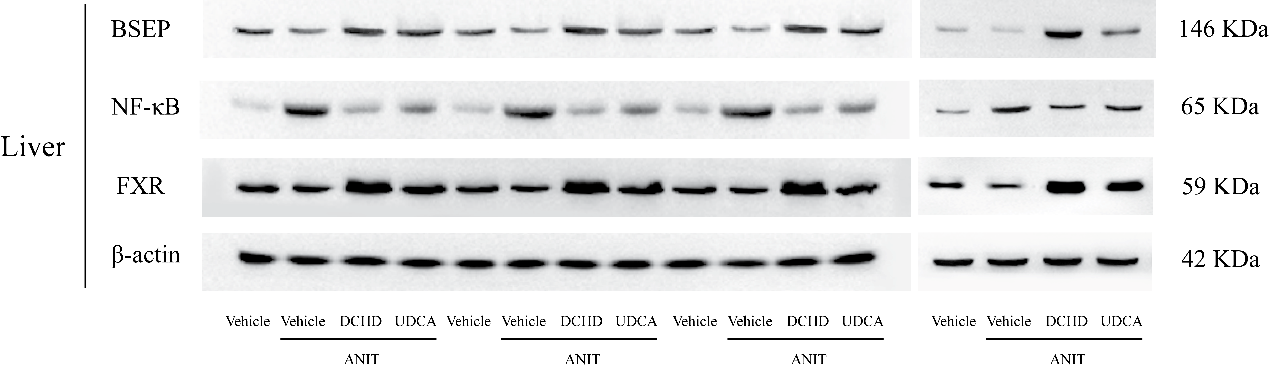


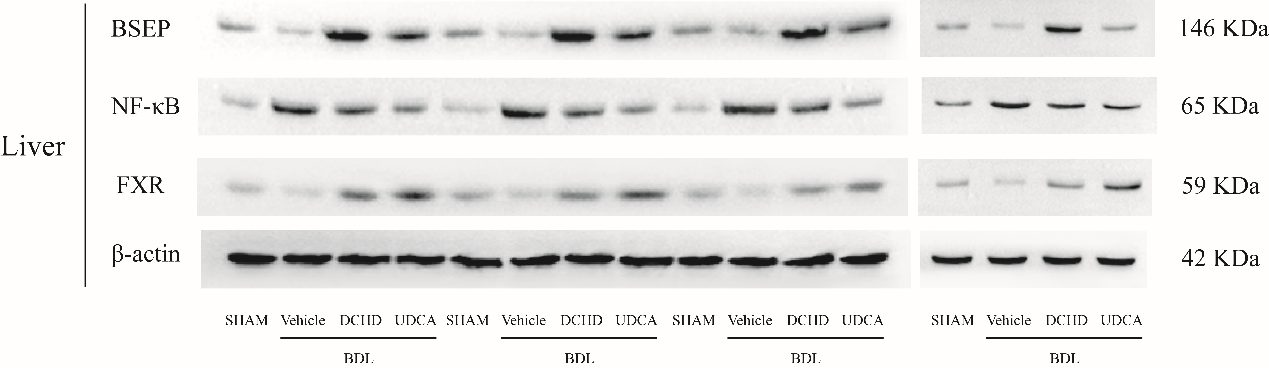


**Supplementary Figure S5**

Western blotting image for the expression of BSEP, NF-κB, FXR in ANIT-induced intrahepatic cholestasis mice and BDL-induced extrahepatic cholestasis mice. Data are presented as mean ± SEM (n=4). ^#^P <0.05, ^##^P < 0.01, ^###^P < 0.001, ^####^P < 0.0001, versus the Vehicle or sham group; ^*^P < 0.05, ^**^P < 0.01, ^***^P < 0.001, ^****^P < 0.0001, versus the ANIT or BDL group; ^△^P < 0.05, ^△△^P < 0.01, ^△△△^P < 0.001, ^△△△△^P < 0.0001, versus the UDCA group.

**2. Supplementary Table**

| **Supplementary Table S1. Experiment regents list** | | |
| --- | --- | --- |
| Regents | Lot number | Manufacture |
| ALT | H556, H551 | SHINO-TEST CORPORATION, Japan |
| AST | 1557, B548 | SHINO-TEST CORPORATION, Japan |
| ALP | C549, D551 | SHINO-TEST CORPORATION, Japan |
| TBA | 150803, 151019 | SHINO-TEST CORPORATION, Japan |
| TBIL | KG970, KG972 | SHINO-TEST CORPORATION, Japan |
| DBIL | KH668, KH672 | SHINO-TEST CORPORATION, Japan |
| γ-glutamyl transpeptidase ELISA assay kits | EK3281 | SAB Signalway Antibody, USA |
| Alpha-naphthyl isothiocyanate | N4525 | SIGMA, USA |
| Ursodeoxycholic  Acid Capsules | H20150398 | Losan Pharma GmbH |
| Isoflurane | R510-22-16 | RWD Life Science Co., Ltd. |
| Edible olive oil | 20200310 | Sinopharm Chemical Reagent Co., Ltd. |
| Chloroform | 20200117 | Sinopharm Chemical Reagent Co., Ltd. |
| Isopropyl alcohol | 20200401 | Sinopharm Chemical Reagent Co., Ltd. |
| Anhydrous ethanol | 20200513 | Sinopharm Chemical Reagent Co., Ltd. |
| Total RNA extraction kit | 9109 | TaKaRa |
| Reverse Transcription Kit | RP047A | TaKaRa |
| SYBR®Premix Ex Taq TMⅡ | RR820A | TaKaRa |
| RNase free dH_2_O | D2215 | TaKaRa |
| DEPC water | R0021 | Beyotime |
| BCA protein detection kit | P0012S | Beyotime |
| Hypersensitive ECI Chemiluminescence Kit | WB0164 | WellBio |
| PVDF membrane | PVH00010 | Immobilon |

| **Supplementary Table S2. Experiment bile acid standards list** | | | | |
| --- | --- | --- | --- | --- |
| Index | Full name | Abbreviation | Manufacture | Lot number |
| 1 | Cholic acid | CA | SIGMA | C1129 |
| 2 | Chenodeoxycholic acid | CDCA | SIGMA | C9377 |
| 3 | Deoxycholic acid | DCA | Aladdin | H1725076 |
| 4 | Lithocholic acid | LCA | SIGMA | L6250 |
| 5 | Ursodeoxycholic acid | UDCA | Aladdin | E1715044 |
| 6 | Hyodeoxycholic acid | HDCA | Aladdin | K1715026 |
| 7 | Glycocholic acid | GCA | Aladdin | H1520042 |
| 8 | Taurocholic acid | TCA | Aladdin | F1729023 |
| 9 | Glycodeoxycholic acid | GDCA | SIGMA | G9910 |
| 10 | Taurodeoxycholic acid | TDCA | SIGMA | T0875 |
| 11 | Glycochenodeoxycholic acid | GCDCA | Meilune | F1218A |
| 12 | Taurochenodeoxycholic acid | TCDCA | SIGMA | T6260 |
| 13 | Glycochenodeoxycholic acid | GUDCA | SIGMA | BCBM2974V |
| 14 | Tauroursodeoxycholic acid | TUDCA | SIGMA | SLBH9352V |
| 15 | Taurohyodeoxycholic acid | THDCA | SIGMA | T0682 |
| 16 | Taurolithocholic acid | TLCA | SIGMA | T7515 |
| 17 | Glycolithocholic acid | GLCA | SIGMA | SLCD1286 |
| 18 | Glycohyodeoxycholic acid | GHDCA | Steraloids | C0867‐000 |

| **Table S3. The gradient of mobile phase for bile acids detection** | | |
| --- | --- | --- |
| Time（min） | Mobile phase A (%) | Mobile phase B (%) |
| 0-1 | 45→45 | 55→55 |
| 1-9 | 45→20 | 55→80 |
| 9-11.4 | 20→10 | 80→90 |
| 11.4-14 | 10→10 | 90→90 |
| 14-14.1 | 10→45 | 90→55 |
| 14.1-17 | 45→45 | 55→55 |

| **Supplementary Table S4. The parameters of mass spectrometer for bile acids detection** | | | | | |
| --- | --- | --- | --- | --- | --- |
|  | Q1Mass (m/z) | Q3Mass (m/z) | DP (Volts) | CE (Volts) | CXP (Volts) |
| TUDCA | 498.1 | 80.0 | -46.0 | -160.0 | -13.0 |
| TDCA | 498.1 | 79.9 | -81.0 | -143.0 | -11.0 |
| TCDCA | 498.4 | 79.9 | -63.0 | -136.0 | -16.6 |
| TCA | 514.1 | 79.9 | -40.0 | -141.0 | -10.0 |
| GCA | 464.2 | 74.1 | -92.5 | -90.8 | -10.0 |
| GCDCA | 448.2 | 74.0 | -20.7 | -100.0 | -10.0 |
| HDCA | 391.3 | 391.3 | -110.0 | -31.0 | -19.0 |
| CA | 407.4 | 407.4 | -16.0 | -32.0 | -19.0 |
| DCA | 391.1 | 391.1 | -187.0 | -29.0 | -18.0 |
| CDCA | 391.3 | 391.2 | -78.0 | -30.8 | -20.0 |
| UDCA | 391.3 | 391.1 | -193.0 | -35.0 | -20.0 |
| GDCA | 448.3 | 74.0 | -160.0 | -84.0 | -10.0 |
| TLCA | 482.3 | 80.0 | -91.0 | -145.0 | -9.0 |
| THDCA | 498.3 | 79.9 | -42.0 | -161.0 | -12.0 |
| LCA | 375.3 | 375.3 | -66.0 | -24.0 | -10.0 |
| GUDCA | 448.3 | 74.1 | -43.0 | -82.5 | -8.4 |
| IS | 319.0 | 191.0 | -107.0 | -36.0 | -19.0 |
| GLCA | 432.2 | 74.0 | -120.0 | -68.0 | -4.0 |
| GHDCA | 448.3 | 74.3 | -43.0 | -82.5 | -8.4 |

| **Supplementary Table S5. The bile acids profile in the serum, liver, and bile of intrahepatic cholestasis mice.** | | | | | |
| --- | --- | --- | --- | --- | --- |
|  | Name | Vehicle | Vehicle + ANIT | DCHD 12 g/kg+ ANIT | DCHD 24 g/kg+ ANIT |
| Serum | CA | 771.95±404.89 | **47685.00±28969.46^####^** | **7881.00±3054.10^****^** | **6720.00±2682.10^****^** |
|  | CDCA | 12.63±6.41 | **249.10±106.70^####^** | **119.60±27.70^****^** | **100.40±15.12^****^** |
|  | DCA | 117.80±98.84 | **418.55±110.22^####^** | **50.63±27.72^****^** | **85.68±27.97^****^** |
|  | GCA | 2.11±0.79 | **848.00±314.28^####^** | **329.25±141.23^****^** | **254.50±159.99^****^** |
|  | GDCA | 13.85±0.56 | **100.53±15.98^###^** | **151.10±61.08^*^** | **346.10±66.16^****^** |
|  | HDCA | 9.22±1.18 | **237.48±96.79^####^** | **36.03±17.93^****^** | **37.33±16.10^****^** |
|  | LCA | 2.11±0.79 | **848.00±314.28^####^** | **329.25±141.23^****^** | **254.50±159.99^****^** |
|  | TCA | 83.35±30.11 | **479450.00±213546.27^####^** | **203600.00±21578.83^****^** | **209825.00±48587.76^****^** |
|  | TCDCA | 14.13±4.15 | **2239.75±632.39^####^** | **4130.00±662.77^****^** | **4580.00±1579.69^****^** |
|  | TDCA | 2.11±0.79 | **848.00±314.28^####^** | **329.25±141.23^**^** | **254.50±159.99^**^** |
|  | THDCA | 32.09±11.05 | **2334.50±1139.28^####^** | **1664.25±1001.41** | **1128.50±155.98^**^** |
|  | TLCA | 514.65±159.76 | **24320.00±9422.96^####^** | **5730.50±3050.01^****^** | **4873.75±2709.47^****^** |
|  | TUDCA | 2.11±0.79 | **848.00±314.28^##^** | **329.25±141.23^**^** | **254.50±159.99^****^** |
|  | UDCA | 14.93±3.75 | 699.00±388.20 | **1567.50±368.62^**^** | 1725.75±704.70 |
|  | TMCA | 218.45±102.40 | **461400.00±146220.03^####^** | **115575.00±39045.53^****^** | **187900.00±88093.91^****^** |
|  | a-MCA | 82.60±27.19 | **24047.50±12035.59^####^** | **3915.50±2110.06^****^** | **3374.25±1317.35^****^** |
|  | b-MCA | 156.95±90.80 | **47375.00±16757.58^####^** | **4254.00±2537.52^****^** | **3068.00±1355.62^****^** |
|  | GUDCA | 5.02±0.43 | 94.08±17.67 | 32.66±25.72 | 17.80±2.26 |
| Liver | CA | 779.95±329.03 | **4405.00±753.57^####^** | **5300.00±794.55^*^** | 4973.00±1402.90 |
|  | CDCA | 3615.00±1227.43 | **1270.50±579.77^###^** | **2429.00±1255.15^*^** | 2152.50±1004.25 |
|  | DCA | 133.25±56.67 | **269.55±72.53^###^** | 248.40±44.72 | **177.10±64.15**** |
|  | GCA | 46.76±22.84 | **475.85±109.67^####^** | 428.60±110.46 | **277.30±120.35***** |
|  | HDCA | 1194.00±334.19 | 872.05±577.80 | 1297.50±694.86 | 1532.50±971.92 |
|  | LCA | 892.50±61.35 | **2581.00±423.57^####^** | **1679.00±228.25^****^** | **1175.50±276.55****** |
|  | TCA | 28987.50±11905.28 | **1379500.00±262414.61^####^** | 1299000.00±87053.35 | **1001000.00±292892.96***** |
|  | TCDCA | 506.70±327.72 | **1339.50±566.62^#^** | **2242.50±1166.08*** | 1709.00±500.32 |
|  | TDCA | 2609.38±651.11 | **402.88±267.81^###^** | **1931.25±1688.98*** | **2253.13±1476.65**** |
|  | THDCA | 1599.50±376.45 | 1312.00±840.16 | 2042.50±1177.53 | **3269.00±1911.30**** |
|  | TLCA | 28.14±5.98 | **59.83±16.71^####^** | 56.85±4.12 | **39.79±12.50**** |
|  | TUDCA | 34.72±20.10 | **867.00±385.82^##^** | 1102.50±606.18 | **2664.00±1979.04**** |
|  | UDCA | 1184.50±526.68 | 763.90±449.99 | 1020.00±358.71 | 1192.65±573.29 |
|  | TMCA | 5422.50±190.62 | **279450.00±103490.17^####^** | **171950.00±45719.86**** | **144575.00±74423.92***** |
|  | α-MCA | 463.10±168.44 | **2681.50±638.11^####^** | **1599.00±166.88****** | **1240.50±488.02****** |
|  | β-MCA | 2121.50±1258.64 | **14550.00±1196.90^####^** | 13300.00±883.37 | **11945.00±2318.61****** |
| Bile | CA | 1475.50±810.06 | 1294.00±590.57 | **3468.00±1349.03^**^** | **3748.00±1917.02^**^** |
|  | CDCA | 579.75±416.68 | **92.65±40.69^##^** | **476.70±131.28^**^** | **828.50±337.55^****^** |
|  | DCA | 379.85±148.62 | **150.40±57.32^#^** | **406.80±205.66^*^** | **563.80±348.48^****^** |
|  | GCA | 9060.00±4521.91 | **1976.00±1220.21^##^** | **11945.00±5239.71^****^** | **9484.50±4013.34^***^** |
|  | GLCA | 783.25±275.93 | 1373.50±682.40 | 1602.00±1488.14 | 1281.05±832.91 |
|  | HDCA | 172.05±105.94 | 142.05±82.49 | 143.50±52.18 | 221.55±123.32 |
|  | LCA | 9060.00±4521.91 | 1976.00±1220.21 | 11945.00±5239.71 | 9484.50±4013.34 |
|  | TCA | 7904250.00±3821846.44 | 7665000.00±5186988.01 | **19910000.00±4123618.38^****^** | **14110000.00±4578945.61^**^** |
|  | TCDCA | 382825.00±266602.46 | **141000.00±117143.92^#^** | **579600.00±234335.44^***^** | 275100.00±281452.54 |
|  | TDCA | 9060.00±4521.91 | **1976.00±1220.21^##^** | 11945.00±5239.71 | 9484.50±4013.34 |
|  | THDCA | 282400.00±158997.72 | 260400.00±90761.85 | 276800.00±125536.65 | 335900.00±175510.85 |
|  | TLCA | 135.58±41.59 | **30.17±24.30^####^** | **100.25±58.03^**^** | **112.25±52.52^****^** |
|  | TUDCA | 9060.00±4521.91 | **1976.00±1220.21^#^** | **11945.00±5239.71^*^** | **9484.50±4013.34^*^** |
|  | UDCA | 322250.00±207937.65 | 88796.50±122520.31 | 358750.00±117516.48 | **340625.00±286101.71^*^** |
|  | TMCA | 13497500.00±4768149.54 | 7886500.00±5065664.33 | **17815000.00±6209557.15^**^** | 12540000.00±6680256.62 |
|  | α-MCA | 1255.50±729.87 | **448.20±189.13^##^** | **1092.90±416.37^*^** | 841.45±413.06 |
|  | β-MCA | 3309.25±1817.99 | **1270.00±805.24^#^** | **3040.00±1863.33^*^** | 2694.50±1428.29 |

| **Supplementary Table S6. The bile acids profile in the serum, liver, and bile of extrahepatic cholestasis mice.** | | | | | | |
| --- | --- | --- | --- | --- | --- | --- |
|  | Name | SHAM | Vehicle + BDL | DCHD 12 g/kg+ BDL | DCHD 24 g/kg+ BDL | DCHD 48 g/kg+ BDL |
| Serum | CA | 203.60±103.47 | **172.54±100.55** | **60.98±49.69^**^** | **110.40±59.26** | **46.85±24.81^***^** |
|  | CDCA | 22.35±7.86 | **33.48±8.42^##^** | **21.93±5.96^**^** | 28.76±6.72 | **25.98±9.34^*^** |
|  | DCA | 162.80±59.19 | **64.16±17.23^####^** | 55.14±11.59 | 63.24±12.66 | **37.66±2.42^*^** |
|  | GCA | 2.49±0.97 | **650.00±354.89^####^** | **157.13±144.77^****^** | 448.12±317.32 | **246.46±133.82^***^** |
|  | HDCA | 68.60±20.47 | **30.67±21.91^####^** | 23.78±18.53 | 29.77±11.10 | 23.34±13.95 |
|  | TCA | 214.40±45.44 | **395000.00±66300.66^####^** | **253360.00±126668.06^***^** | **307940.00±103042.35^*^** | **159160.00±75671.15^****^** |
|  | TCDCA | 17.08±8.31 | **19936.00±5524.90^####^** | **4412.00±1416.70^****^** | **9062.00±4751.96^****^** | **6610.00±7171.56^****^** |
|  | TDCA | 43.84±11.51 | **203.52±125.89^####^** | **88.08±46.54^**^** | 145.96±102.73 | **83.94±42.18^**^** |
|  | THDCA | 46.55±25.49 | **733.40±314.79^####^** | 694.60±379.11 | 501.64±326.26 | 557.60±466.73 |
|  | TLCA | 0.15±0.02 | **3.91±1.46^####^** | **1.82±0.30^****^** | 3.21±1.12 | **2.24±0.82^***^** |
|  | TUDCA | 18.28±4.14 | **6474.00±2055.15^####^** | **1689.40±323.91^****^** | **3328.40±1733.46^****^** | **1669.80±620.62^****^** |
|  | UDCA | 49.58±17.46 | 46.12±10.10 | **61.58±9.16^**^** | **58.34±15.23^*^** | **63.08±8.72^**^** |
|  | TMCA | 200.70±31.98 | **331800.00±38267.48^####^** | **201540.00±78350.50^****^** | **245880.00±62988.62^***^** | **147860.00±73268.22^****^** |
|  | aMCA | 59.52±16.79 | **289.48±53.31^####^** | **172.50±84.50^***^** | **173.70±87.54^***^** | **74.80±75.95^****^** |
|  | bMCA | 148.32±89.87 | **2017.80±407.29^####^** | **1360.20±482.69^**^** | **996.00±609.74^****^** | **798.32±661.01^****^** |
|  | GCDCA | 0.88±0.17 | **4.26±1.16^####^** | **4.03±1.16^****^** | **3.90±1.18^****^** | **4.54±0.81^****^** |
|  | GDCA | 2.10±0.51 | **12.86±3.39^####^** | 12.25±2.17 | 12.00±2.86 | 11.92±3.01 |
|  | GUDCA | 2.01±0.30 | **9.51±1.43^####^** | 8.79±1.22 | 9.42±0.72 | 10.07±1.15 |
| Liver | CA | 1132.40±694.42 | **1243.60±349.61** | **2632.40±429.63^****^** | **2039.20±935.98^*^** | **1941.20±796.21^*^** |
|  | CDCA | 3539.60±603.90 | **2396.00±289.70^##^** | **4003.20±879.01^****^** | **3225.20±992.90^*^** | **3512.80±905.48^**^** |
|  | GCA | 384.12±198.54 | **1111.70±415.94^####^** | 866.80±240.75 | **697.72±309.33^**^** | **510.64±279.92^****^** |
|  | HDCA | 2947.20±1094.18 | **643.80±381.38^####^** | 1264.50±767.71 | 1118.84±950.07 | 721.44±583.25 |
|  | TCA | 46676.00±25987.96 | **616000.00±89522.19^####^** | 529600.00±118200.43 | **412800.00±108080.79^****^** | **382040.00±108845.16^****^** |
|  | TCDCA | 2432.80±945.86 | **22516.00±9540.08^####^** | **15452.00±6888.63^*^** | **12516.00±5704.94^**^** | **9678.40±6500.38^****^** |
|  | TDCA | 8984.00±2919.68 | **1750.72±1442.37^####^** | **4326.80±2440.07^*^** | **5141.20±2372.19^**^** | **4445.40±3070.95^*^** |
|  | THDCA | 1808.72±1612.72 | **756.64±725.95** | **3283.00±2244.89^**^** | **2546.00±1124.00^*^** | **3799.20±2807.18^***^** |
|  | TLCA | 73.20±2.17 | **71.64±1.40** | **77.80±3.83^****^** | **74.76±2.68^*^** | 71.80±3.58 |
|  | TUDCA | 125.98±58.91 | **3684.80±1986.59^##^** | **10208.00±3945.64^****^** | **9064.00±3087.71^***^** | **7466.80±3814.28^**^** |
|  | UDCA | 1768.00±481.42 | **726.60±423.17^#^** | **5070.00±1777.79^****^** | **2180.40±1367.73^**^** | **1865.20±607.52^*^** |
|  | TMCA | 4256.00±2576.71 | **511320.00±116805.49^####^** | **289240.00±92595.07^****^** | **329520.00±72928.29^****^** | **151804.00±90023.84^****^** |
|  | α-MCA | 789.36±323.18 | **1750.80±330.61^####^** | **946.40±330.97^****^** | **979.20±387.88^****^** | **1073.20±492.66^***^** |
|  | β-MCA | 3927.20±1206.43 | **11356.00±2020.12^####^** | **7905.60±2972.94^**^** | **7408.00±2800.19^***^** | **3581.20±2296.56^****^** |
|  | GCDCA | 5.41±1.93 | 7.82±3.11 | 8.51±2.60 | 7.42±4.05 | 7.12±3.24 |
|  | GDCA | 47.51±10.41 | **24.29±3.92^####^** | **36.83±8.53^**^** | 31.29±8.19 | **32.55±8.96^*^** |
|  | GUDCA | 11.40±1.70 | 12.84±2.07 | 11.91±3.49 | 14.31±3.37 | 13.02±4.11 |
|  | GHDCA | 1.48±0.12 | **1.37±0.11** | **1.65±0.33^*^** | 1.39±0.09 | 1.59±0.51 |
|  | DCA | 461.60±13.62 | **790.00±120.83^####^** | **527.20±48.87^****^** | **462.80±31.39^****^** | **470.80±30.70^****^** |
| Bile | CA | 61920.00±20373.45 | **1397.00±1105.72^####^** | 13078.40±10624.89 | 12996.40±13399.65 | 12836.00±13000.64 |
|  | CDCA | 4852.00±1486.54 | **269.16±218.40^####^** | 746.36±506.38 | 488.68±363.52 | **1023.08±916.50^*^** |
|  | GCA | 157960.00±65433.52 | **13505.20±10546.38^####^** | 30952.00±16177.55 | 32140.00±23765.61 | **38620.00±18549.46^*^** |
|  | HDCA | 10742.00±7284.32 | **294.31±235.58^####^** | **3864.00±3182.26^*^** | 583.80±324.31 | 1172.04±1689.64 |
|  | TCA | 9116000.00±1422636.05 | **6980000.00±1480810.59^#^** | **10376000.00±1356623.26^***^** | **10536000.00±3009744.18^***^** | **9552000.00±2400355.53^**^** |
|  | TCDCA | 1432800.00±580900.60 | **347360.00±148237.43^####^** | 517400.00±241171.53 | 447260.00±322396.03 | 400480.00±164409.12 |
|  | TDCA | 1681800.00±637242.29 | **57605.60±58743.50^####^** | **324600.00±191929.73^*^** | 435948.00±417224.27 | 248600.00±186799.02 |
|  | THDCA | 1388800.00±1002151.11 | **135284.00±107539.39^####^** | 387000.00±233500.23 | 303080.00±273592.92 | 300520.00±267417.15 |
|  | TLCA | 1371.00±419.26 | **62.67±35.33^####^** | **234.56±124.66^*^** | 265.05±219.53 | 275.92±290.00 |
|  | TUDCA | 1672200.00±506063.19 | **172600.00±125554.17^####^** | **756840.00±386104.90^**^** | **611560.00±471039.11^*^** | **460160.00±228414.69^*^** |
|  | UDCA | 4736.00±1812.40 | **199.64±93.03^####^** | **2554.00±1364.66^***^** | 670.76±298.71 | 1015.84±1641.93 |
|  | TMCA | 9862000.00±904234.48 | **8312000.00±1738842.53** | **11116000.00±1621571.25^**^** | **10792000.00±2992066.55^*^** | **10608000.00±3168107.74^*^** |
|  | aMCA | 15896.00±9884.69 | **259.84±148.57^####^** | **1929.60±1179.33^*^** | 940.92±1061.59 | 880.08±636.76 |
|  | bMCA | 31398.00±17333.70 | **1087.80±536.52^####^** | **5804.40±2805.96^**^** | **2654.40±671.13^***^** | 1769.20±1117.20 |
|  | GDCA | 3009.40±1949.16 | **322.70±536.97^####^** | 537.00±359.75 | 926.45±916.93 | 654.72±351.92 |
|  | GUDCA | 1647.40±590.43 | **170.56±132.57^####^** | **424.36±186.29^*^** | 499.48±460.10 | 497.36±286.27 |
|  | GHDCA | 126.12±124.54 | **9.11±4.92^####^** | 25.90±14.91 | 19.06±17.63 | 20.94±12.40 |
|  | DCA | 4728.00±1605.16 | **662.40±52.97^####^** | **1157.60±346.67^*^** | **1225.60±496.67^*^** | 1420.80±1468.60 |
|  | GCDCA | 1657.60±861.51 | **514.40±101.55^#^** | 815.60±376.81 | **637.20±209.84^*^** | **3220.40±2148.91^****^** |

| **Supplementary Table S7. Primer sequence for Real-time RT-PCR analysis** | | | |
| --- | --- | --- | --- |
| Primer | Primer sequence (5’-3’) | | Specise |
| FXR | Forward primer | TCCGGACATTCAACCATCAC | Mouse |
|  | Reverse primer | TCACTGCACATCCCAGATCTC |  |
| NF-κB | Forward primer | GAACGATAACCTTTGCAGGC | Mouse |
|  | Reverse primer | TTTCGATTCCGCTATGTGTG |  |
| BSEP | Forward primer | TCTGACTCAGTGATTCTTCGCA | Mouse |
|  | Reverse primer | CCCATAAACATCAGCCAGTTGT |  |
| GAPDH | Forward primer | AGGTCGGTGTGAACGGATTTTG | Mouse |
|  | Reverse primer | GGGGTCGTTGATGGCAACA |  |

| **Supplementary Table S8. Experiment antibody** | | | | |
| --- | --- | --- | --- | --- |
| Antibody |  | Lot number | MW | Manufacture |
| FXR | anti-NR1H4 | AB56902 | 69 kDa | abcam |
| NF-κBp65 | anti-NF-κB p65 | AB112494 | 65 kDa | abcam |
| BSEP | anti-ABCB11 | AB32536 | 59 kDa | abcam |
| β-actin | β-actin (13E5) Rabbit mAb | 4970 | 42 kDa | CST |
| Goat anti-rabbit IgG HRP linked  antibody | | A0216  A0208 |  | Beyotime |
| Horse anti-mouse IgG HRP linked  antibody | | MR-G100 |  | MRbiotech |
